# Phenylpropanoid Amides from *Buteae monospermae* Semen as Hepcidin-Modulating Candidates for Anemia of Inflammation: Network Pharmacology, Deep Learning Prediction, and In Vitro Validation

**DOI:** 10.3390/molecules31214031

**Published:** 2026-11-09

**Authors:** Hongfang Li, Qin Zhang, Liyan Xia, Xiaolin Ding, Rongping Zhang, Bo Hou

**Affiliations:** School of Chinese Materia Medica, Yunnan University of Chinese Medicine, Kunming 650500, China; 16606976271@163.com (Q.Z.); lilyxi2027@163.com (L.X.); 15770374845@163.com (X.D.); zhrpkm@163.com (R.Z.)

**Keywords:** anemia of inflammation, hepcidin, *Buteae monospermae* Semen, phenylpropanoid amides, IL-6/JAK2/STAT3 signaling pathway, network pharmacology, deep learning, HepG2 cells

## Abstract

Anemia of inflammation (AI) is largely driven by interleukin-6 (IL-6)-induced hepcidin overproduction, which disrupts systemic iron homeostasis. *Buteae monospermae* Semen (BMS), the seeds of *Butea monosperma* (Lam.) Kuntze and a traditional Tibetan medicine, has long been used for blood-related disorders, yet its active constituents against AI remain unclear. Here, an integrated strategy combining ADMET screening, network pharmacology, deep learning-based affinity prediction, molecular docking, molecular dynamics simulation, and in vitro validation was applied to 58 BMS-derived compounds. Forty-three candidates with acceptable ADMET profiles were retained, and their predicted targets were enriched in inflammation- and iron metabolism-related pathways, particularly IL-6/JAK2/STAT3 signaling. Two phenylpropanoid amides, compounds **16** and **21**, were prioritized and shown to interact favorably with hepcidin-related regulatory targets. In IL-6-stimulated HepG2 cells, both compounds dose-dependently reduced hepcidin levels (EC_50_ = 2.486 and 14.49 μM), with therapeutic indices of 4.40 and 33.7, respectively. These findings identify BMS-derived phenylpropanoid amides as hepcidin-modulating candidates for AI and demonstrate an effective computational–experimental strategy for discovering bioactive compounds from traditional medicines.

## 1. Introduction

Anemia of inflammation (AI), also known as anemia of chronic disease, is a common form of anemia associated with chronic infections, autoimmune diseases, malignancies, chronic kidney disease, and other persistent inflammatory disorders [1]. It is clinically important because it contributes to fatigue, reduced physical performance, impaired quality of life, and poor disease outcomes in patients with chronic inflammatory conditions [2]. The pathogenesis of AI is closely linked to inflammation-driven disruption of systemic iron homeostasis and iron-restricted erythropoiesis [3]. Among the inflammatory mediators involved, interleukin-6 (IL-6) plays a central role by activating the Janus kinase 2/signal transducer and activator of transcription 3 (JAK2/STAT3) signaling pathway, thereby promoting hepatic expression of hepcidin, a key hormone regulating systemic iron metabolism [4]. Elevated hepcidin induces the internalization and degradation of ferroportin, the only known cellular iron exporter, leading to reduced intestinal iron absorption and impaired iron release from macrophages [5]. As a result, iron becomes unavailable for erythropoiesis despite adequate or increased body iron stores, ultimately contributing to functional iron deficiency and anemia [6]. Current therapeutic strategies for AI mainly involve treatment of the underlying inflammatory disease, administration of erythropoiesis-stimulating agents, and iron supplementation in selected clinical settings; however, their effectiveness may be variable and their application may be restricted by high cost, disease-specific indications, and potential adverse effects [7]. Moreover, these treatments do not consistently correct the upstream dysregulation of hepcidin that links chronic inflammation to iron-restricted erythropoiesis. Therefore, identifying novel bioactive compounds capable of suppressing inflammation-induced hepcidin expression may provide a promising strategy for the development of hepcidin-targeted interventions against AI.

Natural products continue to provide structurally diverse starting points for drug discovery [8]. *Buteae monospermae* Semen (BMS), the dried mature seeds of *Butea monosperma* (Lam.) Kuntze and traditionally known as Maruzi in Tibetan medicine, has been used for blood-related disorders [9]. Previous phytochemical investigations have revealed that BMS contains multiple classes of natural products, including flavonoids and phenylpropanoid amides [10]. These constituents suggest potential pharmacological relevance; however, the specific bioactive compounds responsible for the effects of BMS and their mechanisms of action remain insufficiently understood. In particular, whether BMS-derived compounds can regulate IL-6-induced hepcidin expression has not been systematically investigated. This knowledge gap limits the mechanistic interpretation and further development of BMS-derived bioactive compounds for AI-related applications.

Network pharmacology links compounds, predicted targets, disease genes, and pathways into a unified network, making it a practical way to explore how traditional medicines exert their effects [11]. By integrating compound information, predicted targets, disease-associated targets, and pathway enrichment analyses, this approach can provide a systems-level view of potential pharmacological mechanisms [12]. However, network pharmacology-based predictions may be affected by database bias, incomplete target annotation, and false-positive associations [13]. In recent years, deep learning (DL)-based drug–target interaction prediction has provided an additional computational strategy for identifying potential compound–target relationships by learning molecular representations from compound structures and protein sequences [14]. The integration of DL-based target prediction with network pharmacology may therefore improve the reliability of candidate prioritization and strengthen mechanistic inference. Furthermore, molecular docking and molecular dynamics (MD) simulation can provide complementary structural evidence for ligand–target interactions, supporting the selection of candidate compounds for biological validation [15,16].

Here, we combine network pharmacology, deep learning-based affinity prediction, molecular docking, MD simulations, and IL-6-stimulated HepG2 cell assays to identify hepcidin-modulating compounds among 58 BMS-derived isolates. The prioritized compounds—compound **16** and compound **21**—were evaluated for hepcidin expression and cytotoxicity. The overall workflow is shown in Figure 1.

By integrating network pharmacology, a deep learning-based affinity model, and in vitro validation, this study provides a hypothesis-driven pipeline for discovering natural-product candidates against anemia of inflammation—a clinically prevalent condition with limited therapeutic options.

## 2. Results

### 2.1. Computational Predictions Identify Core Candidate Compounds, Hub Targets, and Enriched Pathways

To systematically investigate the potential pharmacological mechanisms of *Buteae monospermae* Semen (BMS) against anemia of inflammation (AI), an integrated computational strategy was performed by combining compound-related target prediction, disease-related target collection, protein–protein interaction (PPI) network analysis, deep learning (DL)-based target prediction, and functional enrichment analysis. After ADMET-based screening, 43 BMS-derived candidate compounds were retained, and 778 putative compound-related targets were predicted. In parallel, 5173 AI-related disease targets were collected from disease-associated databases. Venn diagram analysis identified 508 overlapping targets between the compound-related targets and AI-related disease targets, which were considered potential therapeutic targets of BMS against AI and were subsequently used for network pharmacology analysis (Figure 2, left panel). In addition, 155 shared targets were obtained from the intersection of the Davis and BindingDB datasets, providing a standardized target set for DL-based compound–target affinity prediction (Figure 2, right panel).

Regarding stereochemistry, compound **16** ([6]-shogaol, (E)-1-(4-hydroxy-3-methoxyphenyl)dec-4-en-3-one) contains a single trans (E) double bond and no chiral center; its geometry was fixed as E in all calculations. Compound **21** (1-(4-hydroxy-3-methoxyphenyl)-5-methoxydecan-3-one) possesses one stereogenic center at C-5; the racemic mixture was used for ADMET screening and docking, with the lowest-energy conformer selected for molecular docking. No additional stereoisomers were separately screened, as the aim of this study was to identify candidate scaffolds rather than to resolve stereochemical activity differences.

The 508 overlapping targets were further subjected to PPI network construction and topological screening to identify hub targets with potential biological importance. As shown in Figure 3A, the initial PPI network was progressively simplified using CytoNCA-based topological parameters, resulting in a core target subnetwork with higher connectivity. The final hub-target set included TP53, EP300, CTNNB1, SMAD3, SRC, CREBBP, STAT3, HAMP, EGFR, and HDAC1. These targets are closely associated with inflammatory regulation, transcriptional control, cellular stress responses, and hepcidin-related iron metabolism, suggesting that BMS may exert its potential anti-AI effects through a multi-target regulatory mechanism.

To further evaluate the topological importance of the hub targets, the top 10 targets were ranked according to degree centrality. TP53 showed the highest degree value, followed by EP300, CTNNB1, SMAD3, SRC, CREBBP, STAT3, HAMP, EGFR, and HDAC1 (Figure 3D). Among these targets, STAT3 and HAMP are particularly relevant to AI pathogenesis. STAT3 is a key transcriptional mediator of inflammatory signaling, whereas HAMP encodes hepcidin, a central regulator of iron restriction during inflammation. The simultaneous identification of STAT3 and HAMP as hub targets supports the biological relevance of the computational screening results. Their inclusion reflects network-level connectivity to iron-metabolism and hepcidin-regulatory pathways identified by GO/KEGG enrichment, rather than a claim of direct erythroid specificity.

GO enrichment analysis was then performed to characterize the biological functions of the prioritized targets. The enriched biological process terms were mainly associated with response to xenobiotic stimulus, cellular response to hormone stimulus, cellular response to nitrogen compound, cellular response to lipid, cellular response to chemical stress, regulation of apoptotic signaling pathway, response to oxidative stress, and positive regulation of apoptotic process (Figure 3B). In the cellular component category, the enriched terms included transcription regulator complex, membrane raft, membrane microdomain, vesicle lumen, endoplasmic reticulum lumen, cytoplasmic vesicle lumen, RNA polymerase II transcription regulator complex, organelle outer membrane, and perinuclear region of cytoplasm. In the molecular function category, the targets were primarily enriched in transcription factor binding, kinase binding, protein kinase binding, oxidoreductase activity, DNA-binding transcription factor binding, transcription coregulator binding, RNA polymerase II-specific DNA-binding transcription factor binding, protein kinase activity, and protein domain-specific binding. These findings indicate that the prioritized targets may participate in cellular stress responses, transcriptional regulation, kinase-related signaling, apoptosis, and oxidative stress-associated biological processes.

KEGG pathway enrichment analysis further revealed that the prioritized targets were involved in multiple inflammation-, metabolism-, and cell survival-related pathways. The representative enriched pathways included pathways in cancer, lipid and atherosclerosis, PI3K–Akt signaling pathway, apoptosis, hepatitis B, human cytomegalovirus infection, AGE–RAGE signaling pathway in diabetic complications, Kaposi sarcoma-associated herpesvirus infection, proteoglycans in cancer, hepatitis C, viral carcinogenesis, EGFR tyrosine kinase inhibitor resistance, and FoxO signaling pathway (Figure 3C). Although several enriched KEGG terms are annotated as disease-specific pathways, they share common regulatory modules related to inflammation, oxidative stress, apoptosis, lipid metabolism, and transcriptional regulation. These biological processes are consistent with the inflammatory and metabolic disturbances involved in AI.

To further visualize the multi-component, multi-target, and multi-pathway regulatory relationships, a BMS–compound–target–pathway–AI network was constructed. As shown in Figure 3E, the network connected BMS, AI, candidate compounds, core targets, and enriched pathways into an integrated pharmacological interaction map. Multiple BMS-derived candidate compounds were connected with hub targets such as TP53, HAMP, STAT3, EP300, HDAC1, SMAD3, JUN, CTNNB1, and RELA. Notably, HAMP and STAT3 were retained as key targets in the network, further supporting their potential relevance to inflammatory signaling and hepcidin regulation. Among the candidate compounds, compound **16** and compound **21** showed strong associations with multiple core targets and occupied important positions in the compound–target–pathway network. Therefore, based on the combined evidence from PPI topology, functional enrichment, DL-based target prediction, and network connectivity, compound **16** and compound **21** were prioritized as core candidate compounds for subsequent molecular docking, molecular dynamics simulation, and in vitro validation.

As presented in Figure 3A,D, systematic network and topological analyses uncovered the core hub targets mediating the anti-inflammatory anemia activity of BMS. Figure 3D further quantifies the connectivity of the top hub genes, confirming the central roles of TP53, EP300, STAT3, and HAMP in the regulatory network. The functional enrichment profiles of these key targets are visualized in Figure 3B,C. GO analysis revealed enrichment in biological processes related to cellular stress responses, transcriptional regulation, and apoptotic signaling, while KEGG pathway analysis identified multiple inflammation- and metabolism-related pathways. The multi-layer BMS–compound–target–pathway–AI interaction network in Figure 3E intuitively demonstrates the holistic pharmacological characteristics of BMS, highlighting the multi-component, multi-target regulatory mechanism underlying its potential therapeutic effect against AI.

### 2.2. Molecular Docking and Molecular Dynamics Simulations Supported the Binding Feasibility and Dynamic Stability of the Selected Compounds

To further evaluate the potential interactions between the prioritized BMS-derived compounds and their predicted targets, molecular docking was performed for the 43 candidate compounds against selected hub targets. The docking score matrix was visualized using hierarchical clustering to compare compound–target binding patterns across the screened targets, including ABCB11, RXRA, HAMP, ELANE, RUNX3, ACHE, MDM2, ALDH1A2, PDK1, and SOD2. As shown in Figure 4A, compounds **16** and **21** clustered together and showed relatively favorable docking-score profiles across multiple targets. Notably, both compounds exhibited measurable binding feasibility with HAMP-related structures, supporting their further evaluation because HAMP encodes hepcidin, a central regulator of iron restriction in anemia of inflammation. The HAMP gene encodes hepcidin, the central systemic regulator of iron homeostasis and the direct effector molecule elevated in anemia of inflammation (AI); it was therefore selected as the most biologically relevant docking target, consistent with the in vitro readout.

The docking heatmap showed the interaction profiles between candidate compounds and hub targets, with compounds **16** and **21** showing comparatively favorable predicted binding poses across multiple targets. Based on their docking performance and the biological relevance of hepcidin regulation, compounds **16** and **21** were further evaluated against two HAMP-related structures (2KEF and 1M4F). The AutoDock Vina scores of the four predicted complexes ranged from −4.7 to −4.4 kcal/mol (Table 1), which are in the moderately favorable range for transient protein–ligand interactions; HAMP–2KEF–compound **16** showed the strongest predicted binding affinity. Structural analysis revealed that compounds **16** and **21** could occupy the predicted binding regions of HAMP-related structures and interact with surrounding residues through non-covalent interactions, including hydrogen bonding and hydrophobic contacts (Figure 4B).

For each complex, the left panel shows the predicted ligand-binding pose within the protein structure, the middle panel highlights key interacting residues and interaction distances, and the right panel presents the corresponding two-dimensional ligand–residue interaction map. We investigated four complexes: 2KEF–compound **16**, 2KEF–compound **21**, 1M4F–compound **16**, and 1M4F–compound **21**. Figure 4 presents the docking profiles of compound **16** and compound **21** bound to HAMP (PDB ID: 2KEF), whereas the interaction diagrams for the two 1M4F complex systems are shown in Figure S3 (Supplementary Materials). All docking poses were generated using AutoDock Vina, and the conformation with the most negative docking score was selected for each complex; lower scores represent stronger predicted binding affinity. Since the obtained docking scores were only moderate, these data merely provide preliminary support for binding feasibility rather than evidence of high-affinity binding. The docking results suggest that both compounds can be accommodated in the binding regions of 2KEF and 1M4F through multiple non-covalent interactions. Figure S1 presents the 2D chemical structures of all 43 candidate compounds alongside their SMILES strings.

As shown in Figure 4F–I, the protein and ligand RMSD curves reached equilibrium after the initial simulation stage, indicating stable binding conformations of compound **16** and **21** within the 2KEF pocket. RMSF analysis (Figure 4H) illustrated low fluctuation of residues surrounding the binding pocket, consistent with stable hydrogen bond interactions maintained throughout the simulation (Figure 4I).

The docking conformations of compounds **16** and **21** bound to HAMP (1M4F) are displayed in Figure S3A–D, which exhibited stable hydrogen bond networks with key interacting residues including HIS3, PHE4, ILE6, CYS7, and MET21.

To further assess whether the docked complexes remained stable under dynamic conditions, 100-ns molecular dynamics (MD) simulations were performed for the four selected systems: 1M4F–compound **16**, 1M4F–compound **21**, 2KEF–compound **16**, and 2KEF–compound **21**. RMSD and RMSF analyses were used to evaluate the global conformational stability and residue-level flexibility of the complexes. As shown in Figure 4, the RMSD trajectories of the protein–ligand systems showed an overall tendency toward equilibration during the simulation period, indicating that the complexes did not undergo substantial structural disruption. RMSF analysis further revealed residue-specific fluctuations, with most regions maintaining relatively stable conformations. These results suggest that the selected protein–ligand complexes could maintain dynamic stability during the simulation process.

RMSD and RMSF calculations were conducted for the four HAMP–ligand complexes (1M4F–16, 1M4F–21, 2KEF–16, 2KEF–21) throughout the 100 ns molecular dynamics simulation. RMSD trajectories reflect global conformational stability of each complex, while RMSF curves quantify flexible regions at the amino acid residue level. Figure 4 displays protein RMSD, ligand RMSD, residue RMSF and hydrogen bond variations for complexes constructed from the 2KEF structure. Corresponding trajectory profiles of the two 1M4F complexes are available in Figure S4 of the Supplementary Materials. Collectively, these trajectory data demonstrated that all tested complexes retained stable overall conformations during simulation. The RMSD of each complex reached a stable plateau within approximately 20–30 ns and fluctuated within a narrow range (±0.1–0.2 nm) thereafter, confirming that the systems remained dynamically stable throughout the 100 ns production run.

### 2.3. In Vitro Validation Confirmed the Hepcidin-Modulating Activity and Cellular Safety of the Prioritized Compounds

Based on computational screening results, compound **16** and compound **21** were selected for in vitro validation. In all cellular assays, experimental ID 1 corresponds to compound **16**, and experimental ID 6 corresponds to compound **21**. Compounds **2**–**5** and **7**–**8** were commercially available structural analogs included for preliminary structure–activity relationship (SAR) evaluation. An IL-6-stimulated HepG2 cell model was used to evaluate the effects of the candidate compounds on hepcidin production. Compound **16** was identified as [6]-shogaol ((E)-1-(4-hydroxy-3-methoxyphenyl)dec-4-en-3-one) by comparison of its NMR and HR-ESI-MS data with literature values [17]. The detailed purity, ^1^H/^13^C NMR, and HR-MS data for compounds **16** and **21** are provided in Table S5 (Supplementary Materials).

As shown in Figure 5A, IL-6 stimulation markedly increased hepcidin secretion compared with the blank control, confirming successful establishment of the inflammatory hepcidin-overexpression model. Among the tested compounds, experimental ID 1 (compound **16**) showed the most pronounced inhibitory effect on IL-6-induced hepcidin production. Experimental ID 6 (compound **21**) also reduced hepcidin levels, although its effect was weaker than that of compound **16**. In contrast, several structural analogs showed limited inhibitory activity or even increased hepcidin levels under the same experimental conditions. These findings suggest that the hepcidin-modulating activity was not a general property of all tested analogs but was more evident for the prioritized phenylpropanoid amide candidates, particularly compound **16**.

Cell proliferation assays were performed to evaluate the concentration ranges suitable for subsequent activity assessment. Figure 5B displays the proliferation curve of HepG2 cells treated with compound **16**; increasing concentrations reduced cell viability at high doses, indicating that dose selection should avoid cytotoxic or growth-inhibitory ranges. Figure 5C shows that compound **21** exhibited no obvious inhibitory effect on HepG2 cell proliferation within the tested concentration range. Together, these results indicated that the observed hepcidin-lowering effects should be interpreted within the corresponding non-toxic concentration window for each compound.

To compare potency and safety, concentration–response analyses were performed for both hepcidin inhibition and cytotoxicity. As shown in Figure 5D,E, compound **16** inhibited IL-6-induced hepcidin production more potently than compound **21**, with EC_50_ values of 2.486 μM and 14.49 μM, respectively. Cell viability assays indicated that compound **16** and compound **21** exhibited CC_50_ values of 10.93 μM and 488.6 μM, respectively, yielding therapeutic indices (TI = CC_50_/EC_50_) of 4.40 and 33.7. These results support compound **16** as the more potent hepcidin modulator, whereas compound **21** showed a wider therapeutic window; both profiles should be considered in follow-up studies.

## 3. Discussion

### 3.1. Summary of the Main Findings

In this study, we combined computational and experimental approaches to identify hepcidin-modulating constituents from *Buteae monospermae* Semen (BMS) for potential application in anemia of inflammation (AI). Starting from 58 experimentally isolated BMS-derived compounds, ADMET-based screening identified 43 candidate compounds with acceptable predicted drug-like and safety-related properties. Subsequent network pharmacology, protein–protein interaction (PPI) analysis, deep learning (DL)-based target affinity prediction, molecular docking, and molecular dynamics (MD) simulations prioritized two phenylpropanoid amides, compound **16** and compound **21**, for biological validation. In an IL-6-stimulated HepG2 cell model, both compounds reduced hepcidin levels, with compound **16** showing markedly stronger activity than compound **21**. These findings suggest that BMS-derived phenylpropanoid amides, particularly compound **16**, may represent promising candidates for further investigation as hepcidin-modulating agents.

Combining network analysis, molecular simulations, and cell-based validation is a pragmatic strategy for natural products, whose effects typically involve several compounds acting on multiple targets [18,19]. The experimental validation step also helped reduce the uncertainty of purely computational predictions and provided functional evidence for the prioritized compounds.

### 3.2. Potential Involvement of the IL-6/JAK2/STAT3–Hepcidin Axis

Hepcidin is a central regulator of systemic iron homeostasis and plays a critical role in the pathogenesis of anemia of inflammation (AI) [20]. Under inflammatory conditions, IL-6 activates the JAK2/STAT3 pathway to promote HAMP transcription, resulting in increased hepcidin production and ferroportin degradation, thereby restricting iron availability for erythropoiesis [21]. Therefore, targeting IL-6-induced hepcidin overexpression represents a potential therapeutic strategy for AI. In this study, computational analyses identified inflammation- and iron metabolism-related networks, with STAT3 and HAMP emerging as key hub targets. Enrichment analyses suggested that BMS-derived compounds may exert multi-target regulatory effects through pathways involved in inflammatory signaling, stress responses, transcriptional regulation, apoptosis, lipid metabolism, and kinase activity. Molecular docking and MD simulations further supported the potential interaction of compounds **16** and **21** with HAMP-related structures, although their binding affinities require experimental validation. Consistently, in vitro assays demonstrated that compounds **16** and **21** significantly attenuated IL-6-induced hepcidin production in HepG2 cells, with compound **16** showing stronger activity and being selected as the primary candidate among the tested phenylpropanoid amides.

### 3.3. Novelty and Pharmacological Implications

BMS has long been used in traditional medicinal systems, yet its bioactive constituents and molecular mechanisms remain incompletely characterized [8,9,10]. In this study, we provide preliminary evidence that phenylpropanoid amides from BMS may contribute to its potential regulatory effects on inflammation-associated disturbances in iron metabolism. To our knowledge, this is the first study to integrate network pharmacology, deep learning-based target prediction, molecular docking and dynamics simulations, and in vitro validation to prioritize BMS-derived phenylpropanoid amides as candidate modulators of hepcidin expression in anemia of inflammation.

Current AI management targets the underlying inflammation or uses erythropoiesis-stimulating agents and iron supplementation, but these do not directly correct inflammation-driven hepcidin overproduction—the event that limits iron mobilization [1,3,5,7,20,21]. Small molecules that attenuate hepcidin induction could therefore complement existing therapy. Compound **16** is of particular interest because it lowered hepcidin more strongly than compound **21** in vitro and was prioritized across all computational filters.

The HepG2 cell data support the biological relevance of compound **16**, but they remain early-stage evidence. Further work is needed to define its upstream targets, structure–activity relationships, pharmacokinetics, safety, and in vivo efficacy in inflammation-associated anemia models.

### 3.4. Preliminary Safety Considerations

Safety is an important consideration in the early prioritization of natural product-derived lead compounds. In this study, predicted toxicity-related endpoints, including hERG inhibition and Ames mutagenicity, were incorporated into the initial ADMET screening. In addition, cell viability assays were performed to determine whether the observed hepcidin-lowering effects occurred within non-cytotoxic or minimally cytotoxic concentration ranges.

The results indicated that compound **16** and compound **21** did not show obvious cytotoxicity at their effective concentrations used for hepcidin inhibition assays. Compound **16** showed stronger hepcidin-lowering activity at a lower concentration (EC_50_ = 2.486 μM) than compound **21** (EC_50_ = 14.49 μM), whereas compound **21** exhibited a wider therapeutic index (TI = CC_50_/EC_50_ = 33.7) than compound **16** (TI = 4.40), suggesting that potency and safety profiles should be balanced in selecting the lead candidate. However, in vitro cytotoxicity assays alone are insufficient to establish safety. Future studies should evaluate broader toxicity profiles, metabolic stability, plasma protein binding, pharmacokinetics, and in vivo tolerability.

### 3.5. Limitations and Future Perspectives

Several limitations should be acknowledged. First, although the integrated computational strategy improved candidate prioritization, network pharmacology and DL-based predictions remain dependent on database quality, target annotation completeness, and model assumptions. Therefore, the predicted compound–target relationships require further experimental confirmation.

Second, the docking scores of the selected HAMP-related complexes were modest. Molecular docking and MD simulations should therefore be regarded as supportive structural evidence rather than definitive proof of direct target engagement. Additional biophysical or biochemical experiments, such as cellular thermal shift assay (CETSA), surface plasmon resonance, isothermal titration calorimetry, or target-specific binding assays, would be useful to confirm direct interactions between the compounds and their putative targets.

Third, the current biological validation was performed only in an IL-6-stimulated HepG2 cell model. Although this model is commonly used to study hepatic hepcidin regulation, it cannot fully reproduce the complexity of AI in vivo. Future studies should evaluate compound **16** in established animal models of inflammation-induced anemia and measure key pharmacodynamic indicators, including serum hepcidin, serum iron, transferrin saturation, ferritin, hemoglobin, and inflammatory cytokines.

Fourth, the downstream mechanism requires deeper validation. In particular, whether compound **16** reduces hepcidin production by inhibiting IL-6/JAK2/STAT3 signaling, by modulating HAMP transcription, or by acting through other upstream regulatory pathways remains unclear. Western blotting or immunofluorescence analysis of phosphorylated STAT3, total STAT3, JAK2 activation, and HAMP mRNA expression would help clarify the mechanism. The inclusion of pathway inhibitors or rescue experiments would further strengthen causal interpretation.

Finally, compound **16** should be further optimized through medicinal chemistry and structure–activity relationship studies. Such optimization may improve potency, selectivity, solubility, metabolic stability, and pharmacokinetic properties. The structural analogs included in the preliminary screening provide an initial basis for this direction, but systematic SAR analysis remains necessary.

Overall, this study identifies BMS-derived phenylpropanoid amides, particularly compound **16**, as promising hepcidin-modulating candidates and provides a foundation for further mechanistic and preclinical investigation in AI.These results support the translational potential of *Butea monosperma*-derived phenylpropanoid amides as natural hepcidin-modulating candidates for anemia of inflammation, and establish a reproducible computational-to-experimental framework that can be extended to other inflammation-driven disorders.

## 4. Materials and Methods

### 4.1. Plant Material

Seeds of *Butea monosperma* (Lam.) Kuntze (Fabaceae; *Buteae monospermae* Semen, BMS) were collected in November 2021 from a farm in Xishuangbanna Dai Autonomous Prefecture, Yunnan Province, China. The plant material was authenticated by Assoc. Prof. Aixue Zuo, and a voucher specimen (No. 2018-1112) has been deposited at the School of Chinese Materia Medica, Yunnan University of Chinese Medicine (Yunnan Key Laboratory of Southern Medicinal Utilization). The isolation and structural characterization of the compounds used in this study, confirmed by ^1^H NMR and MS, were performed as previously described by our group [10]. A chemical fingerprint of the ethyl acetate extract of BMS was obtained by LC–MS/MS analysis, and the total ion chromatogram is presented in Figure S5.

### 4.2. Construction of the Compound Library and AI-Related Target Dataset

#### 4.2.1. Collection of Compounds and ADMET-Based Screening

A total of 58 compounds experimentally isolated from *Buteae monospermae* Semen (BMS) were used to construct the initial compound library. The chemical structures of these compounds were confirmed by ^1^H nuclear magnetic resonance (^1^H NMR) spectroscopy and mass spectrometry (MS) [10]. The compound names, chemical structures, molecular formulas, and SMILES strings are provided in Table S1. Compound information was retrieved from PubChem (https://pubchem.ncbi.nlm.nih.gov/, accessed on 12 October 2026).

To prioritize compounds with favorable drug-like and safety-related properties, an in silico ADMET-based screening strategy was performed. Physicochemical descriptors, including molecular weight, lipophilicity, hydrogen bond donors, hydrogen bond acceptors, and rotatable bonds, were calculated using the RDKit toolkit (2026.03.4) [22]. Toxicity-related endpoints, including human ether-à-go-go-related gene (hERG) inhibition and Ames mutagenicity, were predicted using the admetSAR 2.0 [23]. The screening criteria were based on Lipinski’s rule of five, Veber’s rule, and predicted toxicity classifications, as summarized in Table 2 [24]. The stereoisomers listed in PubChem/TCMSP for compounds **16** and **21** were enumerated and screened through the same ADMET pipeline; the in vitro experiments used the naturally occurring configuration reported for *Buteae monospermae* Semen.

Compounds were retained only when they simultaneously satisfied the physicochemical criteria and were predicted to have low toxicity risk, defined as non-hERG inhibitors and Ames-negative compounds. After ADMET-based screening, 43 compounds were retained as candidate BMS-derived compounds for subsequent target prediction and network pharmacology analyses.

#### 4.2.2. Prediction of Putative Targets of Candidate Compounds

The potential protein targets of the 43 candidate BMS-derived compounds were predicted using multiple target prediction databases, including SwissTargetPrediction, PharmMapper, Similarity Ensemble Approach (SEA), SuperPred, TargetFishing, and the Comparative Toxicogenomics Database (CTD) [12,13,25,26,27,28]. The SMILES strings or structural files of the candidate compounds were used as inputs according to the requirements of each platform. Target prediction was restricted to Homo sapiens when species selection was available.

All retrieved targets were standardized to official gene symbols using the UniProt database (2026_02 release, https://www.uniprot.org/) [29]. Duplicate entries, non-human targets, and targets without valid gene annotations were removed. After integration and deduplication, 778 unique putative targets associated with the BMS-derived candidate compounds were obtained for subsequent network pharmacology analysis.

#### 4.2.3. Collection of AI-Related Disease Targets

Disease-related targets associated with anemia of inflammation (AI) were collected from GeneCards and Online Mendelian Inheritance in Man (OMIM) [30,31]. The following search terms were used: “anemia of inflammation”, “anemia of chronic disease”, “hepcidin”, “hepcidin antimicrobial peptide”, “HAMP”, and “iron homeostasis”. The searches were performed on 1 March 2026. For GeneCards, targets with a relevance score greater than 5 were retained. OMIM-derived targets were manually curated and converted to official gene symbols.All disease-related targets were standardized using UniProt and merged to remove duplicate entries. A total of 5173 AI-related targets were obtained and used for overlap analysis.

#### 4.2.4. Identification of Overlapping Targets Between BMS and AI

The overlapping targets between the 778 BMS-related putative targets and 5173 AI-related disease targets were identified using Venny (version 2.1.0, https://bioinfogp.cnb.csic.es/tools/venny/index.html, accessed on 10 February 2026) [32]. A total of 508 overlapping targets were obtained and considered potential therapeutic targets of BMS against AI. These overlapping targets were used for subsequent protein–protein interaction network construction, functional enrichment analysis, and candidate target prioritization.

### 4.3. Hierarchical Screening and Predictive Analysis from PPI Network Construction to Deep Learning-Based Target Affinity Prediction

To further prioritize potential targets and candidate compounds, a hierarchical screening strategy was applied after the identification of overlapping targets. First, protein–protein interaction (PPI) network analysis was performed to identify hub targets with important topological roles in the biological interaction network. Subsequently, a deep learning (DL)-based compound–target affinity prediction model was used to provide additional molecular-level evidence for potential compound–target interactions. This integrated strategy was designed to combine systems-level target prioritization with DL-assisted interaction prediction, thereby improving the reliability of candidate compound and target selection.

#### 4.3.1. PPI Network Construction and Identification of Key Targets

The 508 overlapping targets identified in Section 4.2.4 were imported into the STRING database to construct a PPI network [33]. The species was restricted to Homo sapiens, and the minimum required interaction score was set to 0.700. Disconnected nodes were removed from the initial network before further analysis.

The resulting PPI network was imported into Cytoscape software (version 3.10.1) for visualization and topological analysis [11]. The CytoNCA plugin was used to calculate key network centrality parameters, including degree centrality, betweenness centrality, and closeness centrality [34]. Hub targets were screened by integrating the three centrality metrics. Briefly, targets whose degree, betweenness centrality and closeness centrality all exceeded their respective median values were defined as core targets. Candidate compounds corresponding to these core targets were then collected from the compound–target dataset to perform subsequent deep learning-based target affinity prediction.

#### 4.3.2. Deep Learning-Based Compound–Target Affinity Prediction Using the MPNN-CNN Model

We used the DeepPurpose compound–target affinity prediction model that combines a message passing neural network (MPNN) for compound SMILES and a convolutional neural network (CNN) for protein sequences. Compound structures were encoded as molecular graphs and protein sequences as embeddings; the two feature vectors were concatenated and passed through fully connected and regression layers to produce a predicted affinity score. The model architecture is shown in Figure 6.

The DeepPurpose MPNN-CNN model was employed with publicly available pretrained weights. Three independent pretrained checkpoints, originally trained on the BindingDB, Davis, and KIBA benchmark datasets [35,36], were used for compound–target affinity prediction in this study; no additional model training or fine-tuning was performed. Compound identifiers, SMILES strings, protein targets, and amino acid sequences were standardized before prediction. After deduplication and target standardization, 155 common targets were retained. Only compound–target interactions with predicted pKd ≥ 6.0 were retained for the subsequent mechanistic exploration. Because these pretrained weights were derived from general drug–target interaction databases rather than iron-specific data, the predictions should be interpreted as ranking evidence rather than absolute binding affinities; the top-ranked candidates (16 and 21) were independently cross-validated by molecular docking, MD simulations, and the HepG2 cell assay.

The model was implemented using PyTorch (2.9.1) [37] and scikit-learn (1.8.0) in Python (3.13.11) [38]. A fixed random seed of 1 was used to ensure reproducibility during inference. The main architectural parameters and the original training hyperparameters of the pretrained MPNN-CNN model are summarized in Table 3.

Using the pretrained MPNN-CNN model, we predicted binding affinity between core candidate compounds from BMS and disease-associated protein targets. The retained compound–target pairs were integrated with the network pharmacology results to further prioritize core targets and candidate compounds.

#### 4.3.3. Functional Enrichment Analysis and Compound–Target–Pathway Network Construction

To uncover the biological roles and signaling cascades of the screened core targets, Gene Ontology (GO) and Kyoto Encyclopedia of Genes and Genomes (KEGG) enrichment analyses were conducted via the online analytical platform Bioinformatics.com.cn. The target list was analyzed with Homo sapiens as the background organism. Enriched GO terms were classified into biological process, cellular component, and molecular function categories. KEGG pathway enrichment analysis was performed to identify signaling pathways potentially involved in the effects of BMS against AI. Enrichment results satisfying *p* < 0.01 and adjusted *p* < 0.05 simultaneously were defined as statistically significant.

A compound–target–pathway network was then constructed and visualized with Cytoscape software (version 3.10.1) [11]. The network integrated candidate compounds, predicted targets, and significantly enriched pathways. Core targets were screened based on topological parameters including degree centrality, betweenness centrality and closeness centrality; targets with all three metrics above the corresponding median values were identified as core targets. Compounds directly associated with the selected core targets were defined as core candidate compounds and were prioritized for molecular docking, molecular dynamics simulation, and experimental validation. The screening rationale for the selected compounds is summarized in Table 4.

### 4.4. Molecular Docking Analysis

Molecular docking was performed to evaluate the potential binding modes and binding affinities between the prioritized candidate compounds and their predicted protein targets. The three-dimensional crystal or solution structures of six core target proteins relevant to anemia of inflammation, including hepcidin antimicrobial peptide (HAMP, two conformers 1M4F and 2KEF), tumor necrosis factor (TNF, 2I47 and 3KMC), signal transducer and activator of transcription 3 (STAT3, 6NJS), and RELA proto-oncogene NF-κB subunit (RELA, 3CBQ), were retrieved from the Protein Data Bank (PDB) [39]. The selected PDB IDs were 1M4F and 2KEF (HAMP), 2I47 and 3KMC (TNF), 6NJS (STAT3), and 3CBQ (RELA). Two distinct NMR-resolved structures (1M4F and 2KEF) of HAMP-encoded hepcidin were retrieved for subsequent molecular dynamics (MD) simulations; these two conformers were selected to represent the alternative disulfide-bonded states identified via integrated network pharmacology and deep learning screening. When multiple structures were available for the same target, the structure with suitable resolution, completeness, and ligand-binding relevance was preferentially selected.

Protein structures were preprocessed with AutoDockTools (version 1.5.7) [40]. Water molecules, non-essential ions, and unrelated heteroatoms were removed unless they were required for maintaining the binding site architecture. Polar hydrogen atoms were added, and Gasteiger charges were assigned. The prepared receptor structures were saved in PDBQT format for docking analysis. The three-dimensional conformations of the 43 candidate compounds derived from BMS were retrieved from the PubChem database or generated via their corresponding SMILES strings [41]. All ligand conformations were protonated under physiological pH where necessary, subjected to energy minimization with the MMFF94s force field, and exported to PDBQT format using AutoDockTools (version 1.5.7) [40]. The three-dimensional structures were retrieved from the RCSB Protein Data Bank (http://www.rcsb.org).

Blind docking simulations were performed using AutoDock Vina (version 1.1.2) [16]. For each target protein, the docking grid box was set to cover the entire protein or the putative ligand-binding region, depending on structural availability and target characteristics. The grid central coordinates and box dimensions are summarized in Table S2. The exhaustiveness value was fixed at 8, and up to 9 binding conformations were generated for each compound–target pair. Docking outputs were recorded as predicted binding free energies in kcal/mol.

All 43 candidate compounds were docked against the six selected target proteins. The resulting compound–target complexes were ranked according to docking score and binding pose plausibility. Complexes showing favorable binding affinity and reasonable intermolecular interactions, including hydrogen bonding, hydrophobic interactions, π–π stacking, and electrostatic interactions, were prioritized for further molecular dynamics (MD) simulation. Based on this screening, four representative ligand–protein complexes, namely Compound **16**–HAMP (1M4F), Compound **21**–HAMP (1M4F), Compound **16**–HAMP (2KEF), and Compound **21**–HAMP (2KEF), were chosen for subsequent molecular dynamics exploration.

The central coordinates and dimensional parameters of docking boxes for all six receptor structures (HAMP-1M4F, HAMP-2KEF, TNF-2I47, TNF-3KMC, STAT3-6NJS, RELA-3CBQ) are summarized in Table S2 (Supplementary Materials). Each box was sized to fully enclose the active pocket of the corresponding protein, enabling comprehensive sampling of binding poses for all candidate compounds.

Complete predicted binding free energies and spatial coordinates of the optimal binding pose for all 43 BMS-derived candidate compounds docked to the six receptors are provided in Table S3 (Supplementary Materials). For every compound–receptor complex, only the lowest-energy conformation (rank = 1) was reserved for downstream analysis, with its binding energy and pose coordinates tabulated.

### 4.5. Molecular Dynamics Simulations

MD simulations were performed to further assess the dynamic stability and interaction patterns of the top-ranked compound–protein complexes obtained from molecular docking. Four compound–protein systems were selected for MD simulation: compound **16** bound to HAMP (PDB ID: 1M4F), compound **16** bound to HAMP (PDB ID: 2KEF), compound **21** bound to HAMP (PDB ID: 1M4F), and compound **21** bound to HAMP (PDB ID: 2KEF).

All simulations were performed using GROMACS software (version 2024.1) [15]. Protein topologies were generated using the CHARMM36m force field [42]. Ligand topology and parameter files were generated using the CHARMM General Force Field (CGenFF) program [43]. Each compound–protein complex was embedded in a cubic box with a 1.0 nm buffer between the solute and box boundaries. Systems were solvated with TIP3P water, neutralized via Na^+^/Cl^−^ counterions, and adjusted to a physiological NaCl concentration of 0.15 M.

Energy minimization was carried out via the steepest descent algorithm until the maximum atomic force dropped below 1000 kJ mol^−1^ nm^−1^. All systems underwent two successive equilibration stages. First, 100 ps NVT equilibration was executed at 300 K with the velocity-rescale thermostat. Subsequently, 100 ps NPT equilibration was conducted at 1 bar using the Parrinello–Rahman barostat. Heavy atoms of protein and ligand were subjected to position restraints throughout the equilibration runs; all restraints were removed prior to production MD simulations.

Production MD simulations were carried out for 100 ns under NPT ensemble conditions at 300 K and 1 bar. The integration time step was set to 2 fs, and trajectory coordinates were recorded every 10 ps for post-simulation analysis. Periodic boundary conditions were applied along all three spatial axes. Long-range electrostatic interactions were calculated via the particle mesh Ewald (PME) method, while all hydrogen-containing covalent bonds were constrained with the LINCS algorithm. The cutoff distances for short-range electrostatic and van der Waals interactions were both defined as 1.0 nm.

Trajectory analyses were performed using built-in GROMACS tools. The root mean square deviation (RMSD), root mean square fluctuation (RMSF), radius of gyration (Rg), solvent-accessible surface area (SASA), and intermolecular hydrogen bond occupancy were calculated to comprehensively evaluate the conformational stability and dynamic binding characteristics of each compound–protein complex. Structural snapshots and key intermolecular interactions were visualized and rendered using open-source PyMOL (version 3.1.0, Open-Source Build).

## 5. Conclusions

In this study, an integrated computational and experimental strategy was used to identify hepcidin-modulating candidate compounds from *Buteae monospermae* Semen (BMS) for potential application in anemia of inflammation (AI). Starting from 58 experimentally isolated BMS-derived compounds, ADMET screening, network pharmacology analysis, deep learning (DL)-based target affinity prediction, molecular docking, and molecular dynamics (MD) simulations were sequentially applied to prioritize candidate bioactive constituents. Two phenylpropanoid amides, compound **16** and compound **21**, were identified as core candidate compounds.

Subsequent in vitro validation in an IL-6-stimulated HepG2 cell model showed that both compounds reduced hepcidin production, with compound **16** exhibiting markedly stronger activity than compound **21**. Neither compound showed apparent cytotoxicity at its effective concentrations. These findings suggest that BMS-derived phenylpropanoid amides, particularly compound **16**, may serve as promising lead candidates for further investigation as hepcidin-modulating agents against AI.

Overall, this study provides preliminary evidence supporting the potential pharmacological relevance of BMS-derived phenylpropanoid amides in inflammation-associated hepcidin dysregulation. It also demonstrates the utility of a “dry-to-wet” strategy integrating computational prediction with experimental validation for the discovery of bioactive compounds from traditional medicines. Future studies should focus on confirming the precise molecular targets, clarifying the involvement of the IL-6/JAK2/STAT3–HAMP axis, and evaluating the in vivo efficacy, pharmacokinetics, and safety profiles of compound **16**. Collectively, the computational predictions (network pharmacology → deep learning ranking → molecular docking → 100 ns MD stability) aligned with the in vitro observation that compounds **16** and **21** reduced hepcidin production in IL-6-stimulated HepG2 cells, providing a coherent dry-to-wet evidence chain for their hepcidin-modulating activity.

## Figures and Tables

**Figure 1:**
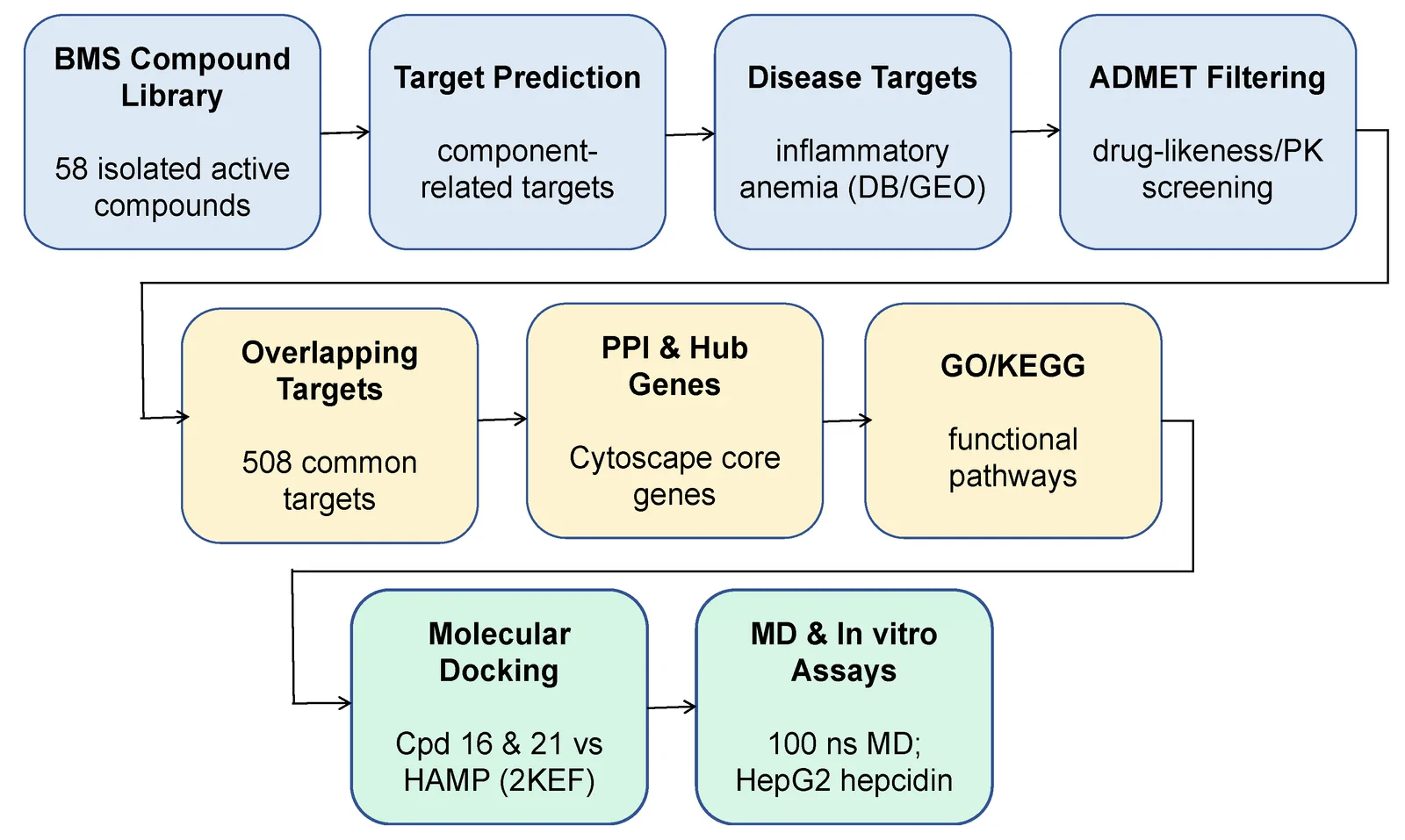
Integrated computational and experimental workflow for identifying BMS-derived hepcidin-modulating candidates against anemia of inflammation. A library of 58 experimentally isolated BMS-derived compounds was subjected to ADMET-based screening, network pharmacology analysis, deep learning (DL)-based drug–target affinity prediction, molecular docking, molecular dynamics (MD) simulation, and in vitro validation. Candidate compounds and targets were prioritized through multi-level computational analyses and subsequently evaluated in an IL-6-stimulated HepG2 cell model. AI, anemia of inflammation; BMS, *Buteae monospermae* Semen; ADMET, absorption, distribution, metabolism, excretion, and toxicity; DL, deep learning; MD, molecular dynamics.

**Figure 2:**
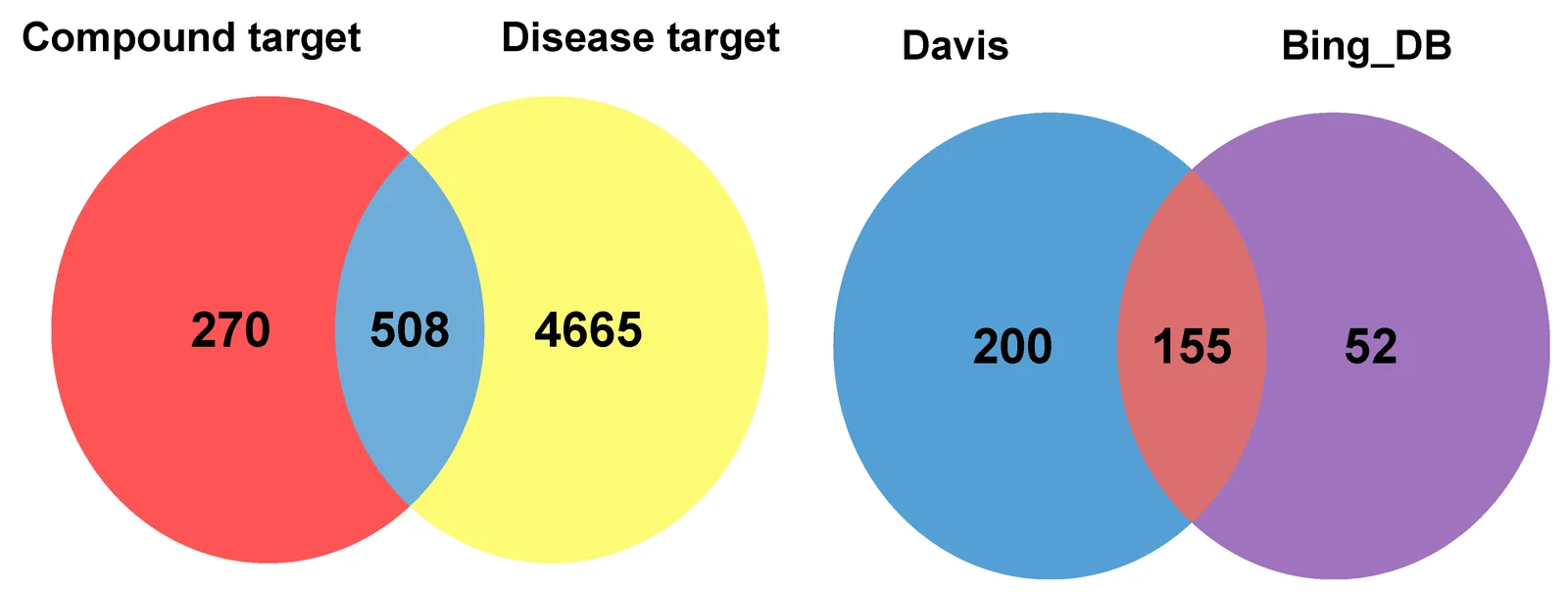
Venn diagram analysis of candidate target screening. The left panel shows the overlap between BMS compound-related targets and AI-related disease targets. A total of 508 overlapping targets were identified and used for subsequent network pharmacology analysis. The right panel shows the overlap of targets derived from the Davis and BindingDB datasets for DL-based compound–target affinity prediction, yielding 155 shared targets. AI, anemia of inflammation; BMS, *Buteae monospermae* Semen; DL, deep learning.

**Figure 3:**
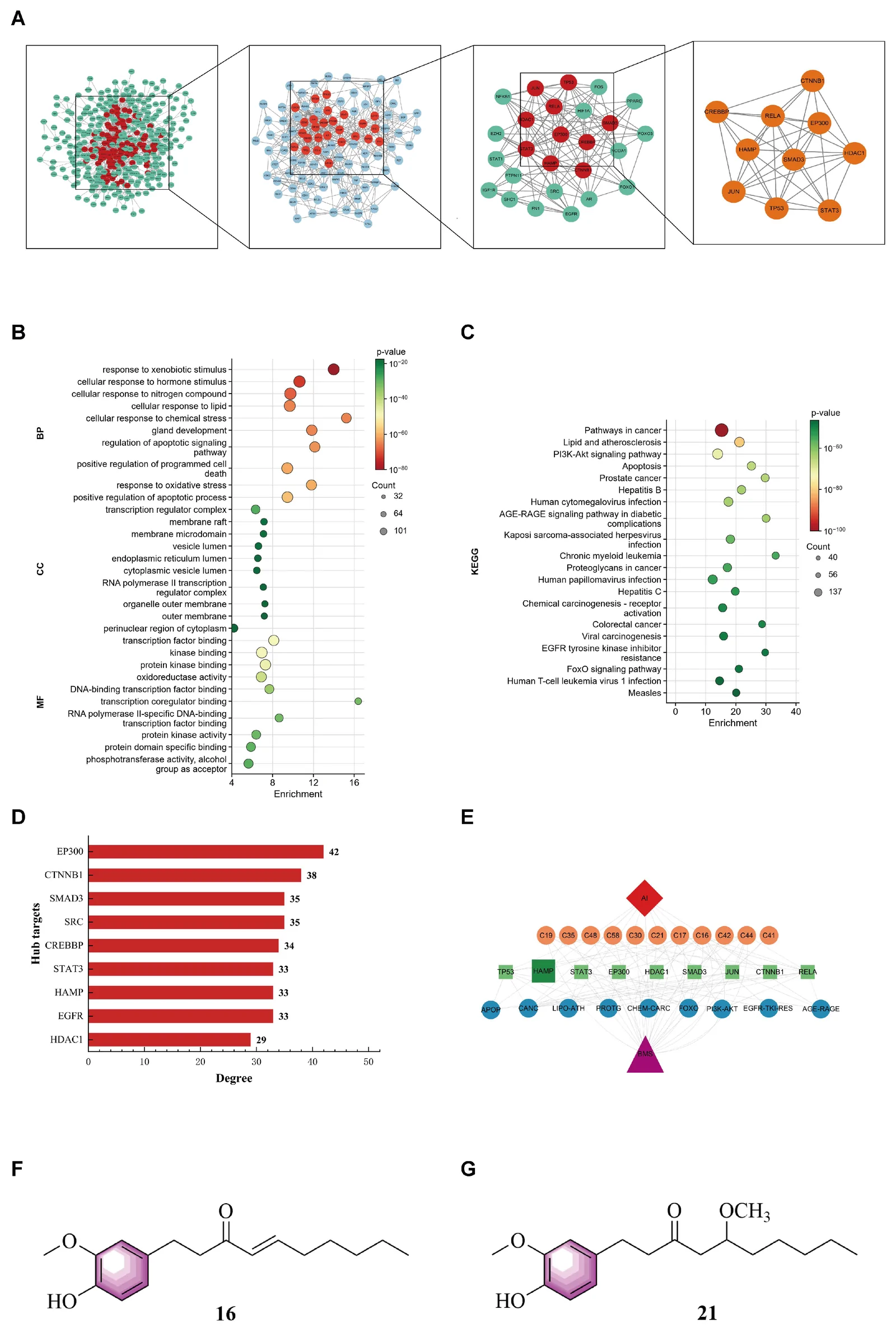
Screening of hub genes and functional enrichment analysis revealing the biological pathways modulated by BMS for the treatment of inflammatory anemia. (**A**) Topological screening of core hub proteins from the PPI network constructed from 508 overlapping target genes. (**B**) Gene Ontology (GO) enrichment analysis of candidate core targets covering biological process, cellular component, and molecular function modules. (**C**) Kyoto Encyclopedia of Genes and Genomes (KEGG) pathway enrichment analysis of core targets. (**D**) Degree distribution of the top 10 hub proteins from the PPI network. (**E**) BMS–compound–target–pathway–AI interaction network. (**F**,**G**) Chemical structures of representative bioactive compounds compound **16** and compound **21**. AI, anemia of inflammation; BMS, Buteae monospermae Semen; GO, Gene Ontology; KEGG, Kyoto Encyclopedia of Genes and Genomes; PPI, protein–protein interaction.

**Figure 4:**
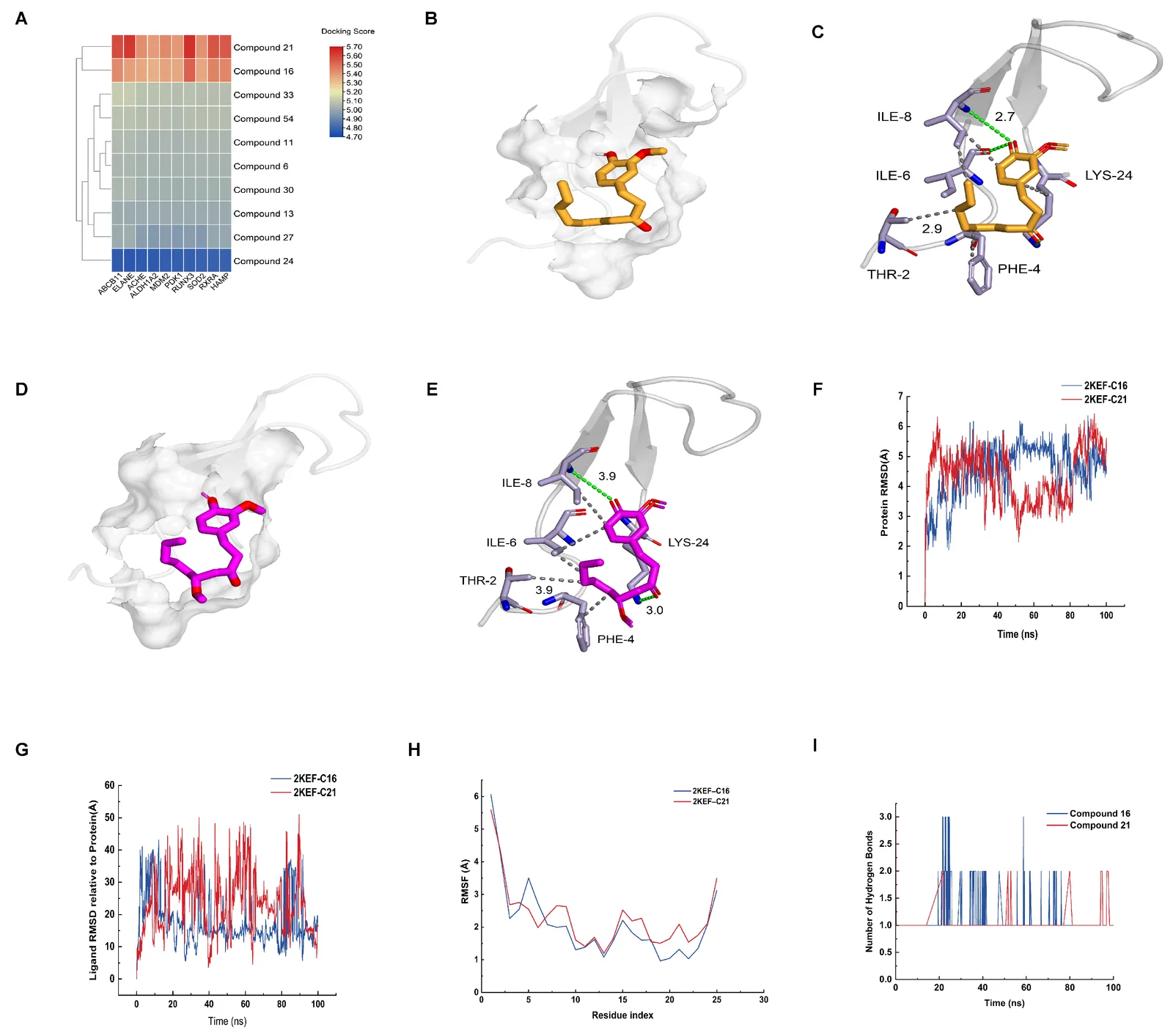
Computational characterization of compound–target binding interactions. (**A**) Hierarchical clustering heatmap of docking scores for screened compound–target pairs. (**B**) Overall binding pocket conformation of compound **16** within HAMP (PDB ID: 2KEF). (**C**) Detailed intermolecular non-covalent interactions between compound **16** and key amino acid residues. (**D**) Overall binding pocket conformation of compound **21** within HAMP (PDB ID: 2KEF). (**E**) Detailed intermolecular non-covalent interactions between compound **21** and key amino acid residues. (**F**) Backbone RMSD curves of the 2KEF-16 and 2KEF-21 complexes over the 100 ns simulation. (**G**) RMSD of ligands referenced to their initial docked poses. (**H**) Residue RMSF represents local structural flexibility. (**I**) Time-dependent changes in intermolecular hydrogen bond counts. RMSD, root-mean-square deviation; RMSF, root-mean-square fluctuation.

**Figure 5:**
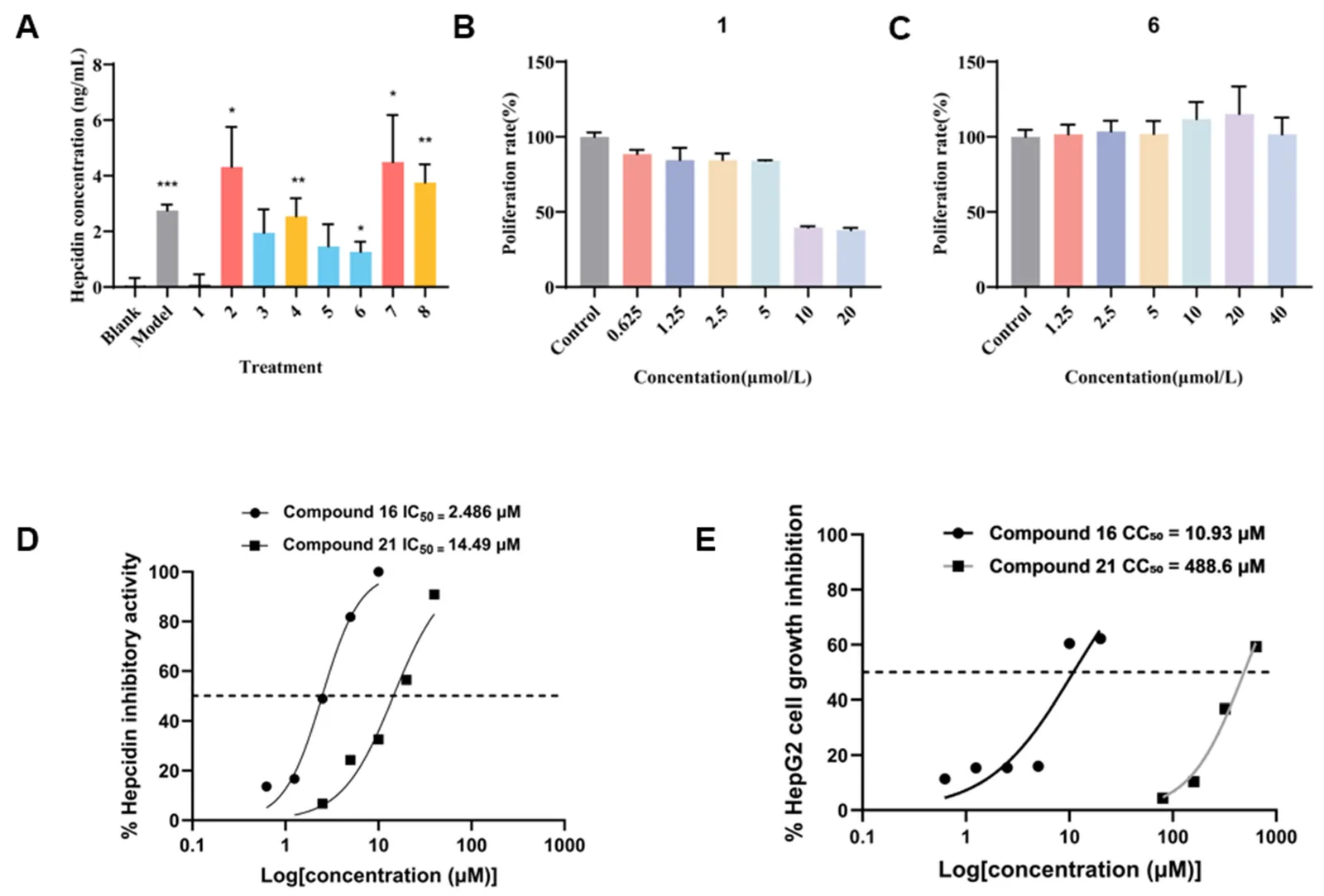
In vitro validation of hepcidin-modulating activity and cytotoxicity of compound **16** and compound **21** in HepG2 cells. (**A**) Preliminary screening of candidate compounds. Quantified hepcidin concentrations of the blank control, IL-6-stimulated model group, and compound-treated groups are shown. Experimental ID 1 represents compound **16**, and experimental ID 6 represents compound **21**; compounds **2**–**5** and **7**–**8** are structural analogs for preliminary SAR analysis. (**B**) Proliferation rates of HepG2 cells treated with gradient concentrations of compound **16**. (**C**) Proliferation rates of HepG2 cells treated with gradient concentrations of compound **21**. (**D**) Concentration–response curves for hepcidin inhibition by compound **16** (closed circles) and compound **21** (open squares). The EC_50_ values of compound **16** and compound **21** for hepcidin inhibition were calculated as 2.486 μM and 14.49 μM, respectively. (**E**) Concentration–response curves for HepG2 cell cytotoxicity. The CC_50_ values of compound **16** and compound **21** were calculated as 10.93 μM and 488.6 μM, respectively. All dose–response data were fitted via nonlinear regression in GraphPad Prism 10.1.2 (GraphPad Software, Boston, MA, USA). Data in (**A**) are presented as mean ± SD. Statistical analysis was performed using one-way ANOVA; *** *p* < 0.001 vs. blank control; * *p* < 0.05 and ** *p* < 0.01 vs. model group. CC_50_, half-maximal cytotoxic concentration; EC_50_, half-maximal effective concentration; SAR, structure–activity relationship.

**Figure 6:**
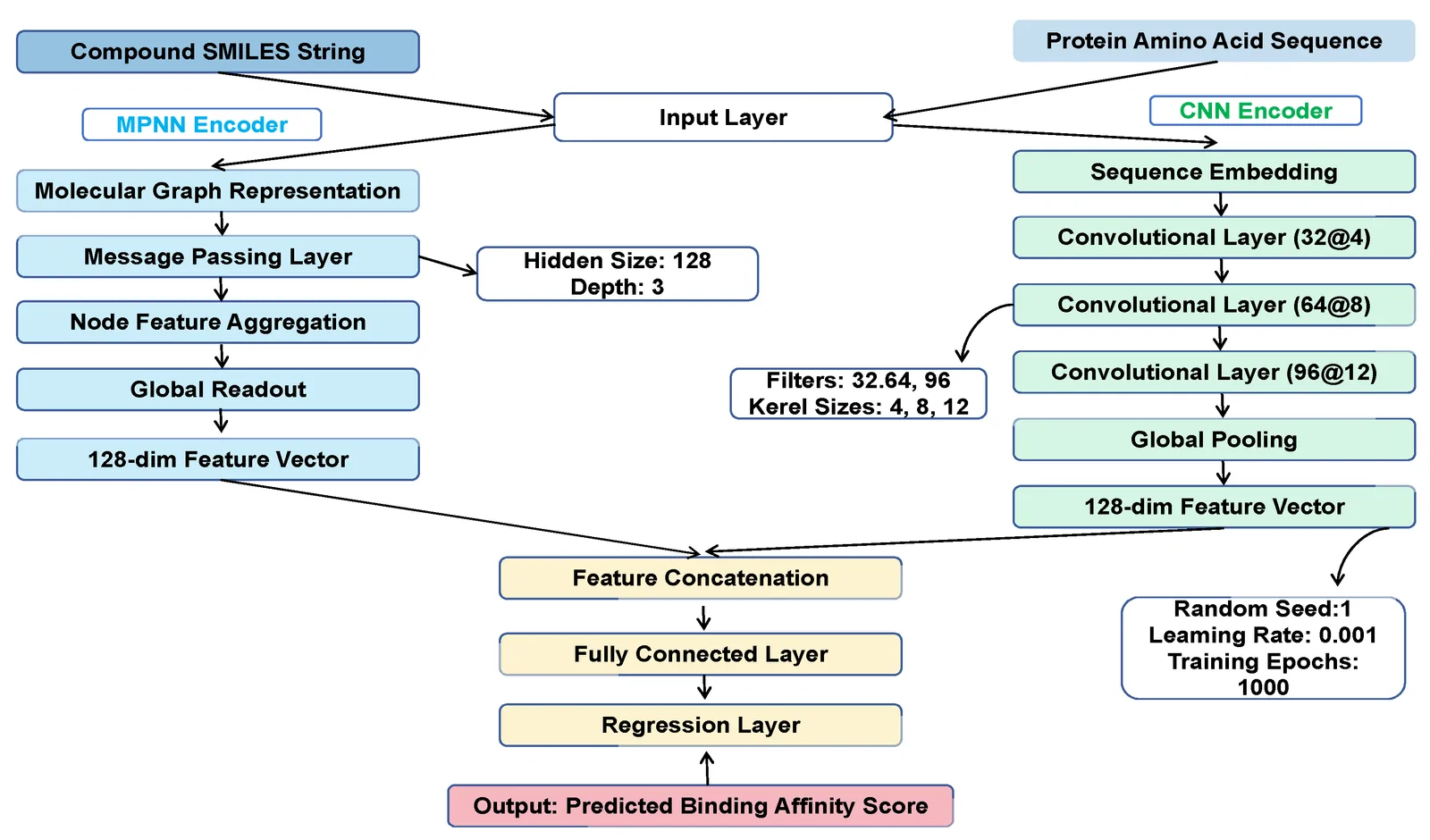
Architecture of the MPNN-CNN model for compound–target affinity prediction. Compound SMILES strings were converted into molecular graph representations and encoded using a message passing neural network (MPNN), whereas protein amino acid sequences were embedded and encoded using a convolutional neural network (CNN). The resulting 128-dimensional compound and protein feature vectors were concatenated and passed through fully connected layers and a regression layer to generate predicted binding affinity scores. Key model parameters included an MPNN hidden size of 128 with a depth of 3, CNN filters of 32, 64, and 96, and kernel sizes of 4, 8, and 12. CNN, convolutional neural network; MPNN, message passing neural network.

**Table 1:** Molecular docking results of selected compounds with HAMP.

Complex	Target Gene	PDB ID	Compound	Binding Energy (kcal/mol)
HAMP-2KEF-C16	HAMP	2KEF	**C16**	−4.7
HAMP-1M4F-C16	HAMP	1M4F	**C16**	−4.5
HAMP-1M4F-C21	HAMP	1M4F	**C21**	−4.5
HAMP-2KEF-C21	HAMP	2KEF	**C21**	−4.4

Note: Binding energy was obtained from AutoDock Vina, and the best-ranked binding mode was selected for each complex. A docking score ≤ −5.0 kcal/mol was considered to indicate favorable binding, following the threshold commonly adopted in AutoDock Vina-based docking studies [16]. Detailed non-covalent interaction analysis using the Protein–Ligand Interaction Profiler (PLIP) identified hydrogen bonds, hydrophobic contacts, and π-interactions involving residues including ILE6, CYS7, PHE4, HIS3, and MET21 (see Figure 4 and Table S4). The detailed interaction profiles of the four systems are provided in Figure 4 and Figure S3 of the Supplementary Materials.

**Table 2:** Preliminary screening criteria for compound-class pharmacological properties based on computational descriptors.

Filter Categories	Calculate Descriptors	Calculation Tools	Filter Criteria	Based on
Pharmacological properties	Molecular weight	RDKit (v2026.03.4) descriptors.ExactMolWt	≤500 Da	Lipinski’s Rule
	Lipophilicity	RDKit (v2026.03.4) Descriptors.MolLogP	≤5	Lipinski’s Rule
	Number of Hydrogen Bond Donors	RDKit (v2026.03.4) Descriptors.NumHDonors	≤5	Lipinski’s Rule
	Number of Hydrogen Bond Acceptors	RDKit (v2026.03.4) descriptors.NumHAcceptors	≤10	Lipinski’s Rule
Molecular Flexibility	Number of Rotatable Bonds	RDKit (v2026.03.4) Descriptors.NumRotatableBonds	≤10	Veber’s Rule
Toxicity Risk	hERG Inhibition	admetSAR 2.0 Prediction Model	Non-inhibitor	Built-in classification within the model
	Mutagenicity (AMES)	admetSAR 2.0 Prediction Model	Negative	Built-in classification within the model

**Table 3:** Architecture of the pretrained MPNN-CNN model.

Category	Parameter	Setting
MPNN	Hidden dimension	128
MPNN	Message passing depth	3
CNN	Filter sizes	32, 64, 96
CNN	Hidden dimension	128

**Table 4:** Screening rationale for compounds selected for experimental validation.

Compound	Degree in Original Network	Retained in Simplified Network	Associated Hub Targets	Experimental Validation
**C16**	Highest	Yes	HAMP, STAT3	Lead compound with SAR analogs
**C35**	High	Yes	HAMP, EP300, JUN	No
**C21**	High	Yes	HAMP, HDAC1	Lead compound with SAR analogs
**C36**	High	No	—	No

Note: Compound **16** and compound **21** were selected as lead candidate compounds based on network topology analysis, hub-target association, DL-based affinity prediction, and structural relevance, as summarized in Table 4. Additional commercially available structural analogs were included for comparison where indicated.

## Data Availability

The original contributions presented in this study are included in the article and Supplementary Material. Further inquiries can be directed to the corresponding authors. The raw molecular docking and molecular dynamics simulation data supporting the conclusions of this article will be deposited in the Figshare repository upon manuscript acceptance.

## Supplementary Materials

The following supporting information can be downloaded at: https://www.mdpi.com/article/10.3390/molecules31214031/s1, Figure S1: Two-dimensional chemical structures of all 43 BMS-derived candidate compounds; Figure S2: Summary of ADMET screening results; Figure S3: Molecular docking interaction profiles (HAMP, 1M4F); Figure S4: Molecular dynamics trajectory analysis (HAMP, 1M4F); Figure S5: LC–MS chemical fingerprint of *Buteae monospermae* Semen extract; Figure S6: NMR spectra of compounds **16** and **21**; Table S1: Chemical information of the 43 ADMET-screened BMS compounds; Table S2: Central coordinates and dimensional parameters of docking grids; Table S3: Predicted binding free energies and optimal binding pose coordinates; Table S4: PLIP protein–ligand interaction analysis (compound **16**/**21** with HAMP); Table S5: Purity, NMR, and HR-MS data for compounds **16** and **21**.

## Abbreviations

ACHE, acetylcholinesterase; ADMET, absorption, distribution, metabolism, excretion, and toxicity; AI, anemia of inflammation; BMS, *Buteae monospermae* Semen; CC_50_, 50% cytotoxic concentration; CHARMM, Chemistry at HARvard Macromolecular Mechanics; CNN, convolutional neural network; CTD, Comparative Toxicogenomics Database; DL, deep learning; EC_50_, half-maximal effective concentration; GO, Gene Ontology; GROMACS, GROningen MAchine for Chemical Simulations; HAMP, hepcidin antimicrobial peptide; hERG, human ether-à-go-go-related gene; IL-6, interleukin-6; JAK2, Janus kinase 2; KEGG, Kyoto Encyclopedia of Genes and Genomes; LC–MS/MS, liquid chromatography–tandem mass spectrometry; MD, molecular dynamics; MPNN, message passing neural network; MPNN-CNN, message passing neural network–convolutional neural network; MS, mass spectrometry; NMR, nuclear magnetic resonance; NPT, constant number, pressure and temperature; OMIM, Online Mendelian Inheritance in Man; PDB, Protein Data Bank; PDBQT, Protein Data Bank with Partial Charges (file format); PPI, protein–protein interaction; RMSD, root-mean-square deviation; RMSF, root-mean-square fluctuation; SMILES, Simplified Molecular Input Line Entry System; STAT3, signal transducer and activator of transcription 3; STRING, Search Tool for the Retrieval of Interacting Genes/Proteins; TI, therapeutic index; TNF, tumor necrosis factor.
