# Supplementary material for: Phenylpropanoid Amides from Buteae monospermae Semen as Hepcidin-Modulating Candidates for Anemia of Inflammation: Network Pharmacology, Deep Learning Prediction, and In Vitro Validation
Source: Molecules. 2026 Nov 9;31(21):4031. doi: 10.3390/molecules31214031 (PMC13764935; doi:10.3390/molecules31214031)
Supplement: Supplementary file 1 [file molecules-31-04031-s001.zip › molecules-4654802-supplementary.pdf]

Phenylpropanoid Amides from Buteae Monospermae Semen as Hepcidin-Modulating Candidates for Anemia of Inflammation: Network Pharmacology, Deep Learning Prediction, and In Vitro Validation

Supplementary Materials

Contents

Table S1. Chemical information of the 43 ADMET-screened BMS compounds.

Table S2. Central coordinates and dimensional parameters of docking grids.

Table S3. Predicted binding free energies and optimal binding pose coordinates.

Table S4. PLIP protein–ligand interaction analysis (compound **16**/21 with HAMP).

Table S5. Purity, NMR, and HR-MS data for compounds 16 and 21.

Note: NMR and HR-MS data were originally reported in Liu QR. Master's thesis: Phenylpropanoid Amides from Buteae Monospermae Semen and their anti-HCoV-OC43 activity. Yunnan University of Chinese Medicine, 2024.

Figure S1. Two-dimensional chemical structures of all 43 BMS-derived candidate compounds.

Figure S2. Summary of ADMET screening results for the 43 compounds.

Figure S3. Molecular docking interaction profiles (HAMP, 1M4F).

Figure S4. Molecular dynamics trajectory analysis (HAMP, 1M4F).

Figure S5. LC–MS chemical fingerprint of BME ethyl acetate extract.

Table S1. Chemical information of the 43 ADMET-screened BMS compounds.

Note: Compound numbers correspond to the original isolation numbering. SMILES strings are provided for cheminformatic reproducibility.

No.	Compound Name	SMILES
1	2-phenyl-4H-furo[2,3-h]chromen-4-one	O=C1C2=C(C(C=CO3)=C3C=C2)OC(C4=CC=CC=C4)=C1
2	3-methoxy-2-phenyl-4H-furo[2,3-h]chromen-4-one	O=C1C2=C(C(C=CO3)=C3C=C2)OC(C4=CC=CC=C4)=C1OC
3	6-methoxy-2-phenyl-4H-furo[2,3-h]chromen-4-one	O=C1C2=C(C(C=CO3)=C3C(OC)=C2)OC(C4=CC=CC=C4)=C1
5	2-(3-methoxyphenyl)-4H-furo[2,3-h]chromen-4-one	O=C1C2=C(C(C=CO3)=C3C=C2)OC(C4=CC=CC(OC)=C4)=C1
6	2-(benzo[d][1,3]dioxol-5-yl)-3,7-dimethoxy-4H-chromen-4-one	O=C1C2=C(C=C(OC)C=C2)OC(C3=CC=C(OCO4)C4=C3)=C1OC
7	2-(benzo[d][1,3]dioxol-5-yl)-3-methoxy-4H-furo[2,3-h]chromen-4-one	O=C1C2=C(C(C=CO3)=C3C=C2)OC(C4=CC=C(OCO5)C5=C4)=C1OC
8	2-(benzo[d][1,3]dioxol-5-yl)-3,6-dimethoxy-4H-chromen-4-one	O=C1C2=C(C=CC(OC)=C2)OC(C3=CC=C(OCO4)C4=C3)=C1OC
9	2-(benzo[d][1,3]dioxol-5-yl)-8,8-dimethyl-2,3-dihydro-4H,8H-pyrano[2,3-f]chromen-4-one	O=C1C2=C(C(C=CC(C)(C)O3)=C3C=C2)OC(C4=CC=C(OCO5)C5=C4)C1
10	8,8-dimethyl-2-phenyl-4H,8H-pyrano[2,3-f]chromen-4-one	O=C1C2=C(C(C=CC(C)(C)O3)=C3C=C2)OC(C4=CC=CC=C4)=C1
11	2-(4-hydroxyphenyl)-3-methoxy-4H-chromen-4-one	O=C1C2=C(C=CC=C2)OC(C3=CC=C(O)C=C3)=C1OC
12	(2R,3R)-3-hydroxy-8,8-dimethyl-2-phenyl-2,3-dihydro-4H,8H-pyrano[2,3-f]chromen-4-one	O=C1C2=C(C(C=CC(C)(C)O3)=C3C=C2)O[C@H](C4=CC=CC=C4)[C@H]1O
13	5,7-dihydroxy-3-(4-hydroxyphenyl)-4H-chromen-4-one	O=C1C2=C(C=C(O)C=C2O)OC=C1C3=CC=C(O)C=C3
16	(E)-1-(4-hydroxy-3-methoxyphenyl)dec-4-en-3-one	OC1=C(OC)C=C(CCC(/C=C/CCCCC)=O)C=C1
17	(1E,4Z)-5-hydroxy-1-(4-hydroxy-3-methoxyphenyl)undeca-1,4-dien-3-one	OC1=C(OC)C=C(/C=C/C(/C=C(O)/CCCCCC)=O)C=C1
19	(1E,3Z)-3-hydroxy-1-(4-hydroxy-3-methoxyphenyl)deca-1,3-dien-5-one	OC1=C(OC)C=C(/C=C/C(O)=C/C(CCCCC)=O)C=C1
21	1-(4-hydroxy-3-methoxyphenyl)-5-methoxydecan-3-one	OC1=C(OC)C=C(CCC(CC(OC)CCCCC)=O)C=C1
24	(S)-4-hydroxy-4-((R,E)-3-hydroxybut-1-en-1-yl)-3,5,5-trimethylcyclohex-2-en-1-one	O=C1CC(C)(C)[C@](/C=C/[C@](O)(C)[H])(O)C(C)=C1

25	(2Z,4E)-5-((1S,3S,5R,8S)-3,8-dihydroxy-1,5-dimethyl-7-oxo-6-oxabicyclo[3.2.1]octan-8-yl)-3-methylpenta-2,4-dienoic acid	O[C@H]1C[C@](C)(C(O2)=O)[C@](/C=C/C(C)=C(C(O)=O)/[H])(O)[C@@]2(C)C1
26	(4S)-4-hydroxy-3,3,5-trimethyl-4-((E)-3-oxobut-1-en-1-yl)cyclohexan-1-one	O=C1CC(C)(C)[C@](/C=C/C(C)=O)(O)C(C)C1
27	(2E,4E)-5-((1R,4S,6S,8R)-6,8-dihydroxy-1,4-dimethyl-2-oxabicyclo[2.2.2]octan-6-yl)-3-methylpenta-2,4-dienoic acid	O[C@@H]1C[C@@](C)(OC2)[C@](/C=C/C(C)=C/C(O)=O)(O)C[C@]12C
28	(S,E)-4-hydroxy-3,5,5-trimethyl-4-(3-oxobut-1-en-1-yl)cyclohex-2-en-1-one	O=C1CC(C)(C)[C@](/C=C/C(C)=O)(O)C(C)=C1
29	(S)-4-hydroxy-3,5,5-trimethyl-4-((R,E)-3-(((2R,3R,4S,5S,6R)-3,4,5-trihydroxy-6-(hydroxymethyl)tetrahydro-2H-pyran-2-yl)oxy)but-1-en-1-yl)cyclohex-2-en-1-one	O[C@H]1[C@H](O)[C@@H](O)[C@H](O[C@@H](/C=C/[C@]2(O)C(C)(C)CC(C=C2C)=O)C)O[C@@H]1CO
30	3,4-dihydroxybenzaldehyde	OC1=C(O)C=C(C([H])=O)C=C1
31	3,4-dihydroxybenzoic acid	OC1=C(O)C=CC(C(O)=O)=C1
32	1-(4-hydroxyphenyl)ethan-1-one	OC1=CC=C(C(C)=O)C=C1
33	4-hydroxybenzaldehyde	OC1=CC=C(C=O)C=C1
35	nicotinamide	NC(C1=CN=CC=C1)=O
36	3H-indole-3-carbaldehyde	O=CC1C=NC2=CC=CC=C21
37	(5S)-4-hydroxy-2-methyl-5-((S)-6-methylhept-5-en-2-yl)cyclohex-2-en-1-one	OC(C=C1C)[C@H]([C@@H](C)CC/C=C(C)\C)CC1=O
38	5,7-dihydroxy-2-methyl-4H-chromen-4-one	OC1=C2C(OC(C)=CC2=O)=CC(O)=C1
39	benzene-1,2,4-triol	OC1=CC=C(O)C=C1O
41	(E)-3-(4-hydroxyphenyl)acrylic acid	OC1=CC=C(/C=C/C(O)=O)C=C1
42	ethyl 4-hydroxybenzoate	OC1=CC=C(C(OCC)=O)C=C1
44	4-hydroxy-3-methoxybenzaldehyde	OC1=C(OC)C=C(C=O)C=C1
48	dibutyl phthalate	O=C(OCCCC)C1=CC=CC=C1C(OCCCC)=O
49	1-(4-methoxy-2,3-dihydrobenzofuran-5-yl)-3-phenylpropane-1,3-dione	O=C(CC(C1=CC=CC=C1)=O)C2=C(OC)C3=C(OCC3)C=C2
51	(Z)-4-((1R,3S,5R,8S)-3,8-dihydroxy-1,5-dimethyl-6-oxabicyclo[3.2.1]octan-8-yl)but-3-en-2-one	C[C@@]1(CO2)[C@](/C=C\C(C)=O)(O)[C@@]2(C)C[C@@H](O)C1
52	2-(2-hydroxyethyl)-3-methoxybenzaldehyde	OCCC1=C(C=O)C=CC=C1OC
53	(E)-3-(4-(2-hydroxyacetoxy)phenyl)acrylic acid	O[C@H]1[C@H](OC2=CC=C(/C=C/C(O)=O)C=C2)[C@@H](O)C(O)[C@@H](CO)O1
54	2-(3,4-dihydroxyphenyl)benzo[d][1,3]dioxole-5-carbaldehyde	OC1=CC=C(C2OC(C=C(C=O)C=C3)=C3O2)C=C1O
55	(2S,3R)-2-(3,4-dihydroxyphenyl)chromane-3,6,7-triol	OC1=CC2=C(C[C@@H](O)[C@H](C3=CC=C(O)C(O)=C3)O2)C=C1O

56	(3R)-2-(3,4-dihydroxyphenyl)chromane-3,5,7-triol	OC1=CC2=C(C[C@@H](O)C(C3=CC(O)=C(O)C=C3)O2)C(O)=C1
58	ethyl (E)-(3-(4-hydroxyphenyl)acryloyl)-L-tyrosinate	OC1=CC=C(C[C@H](NC(/C=C/C2=CC=C(O)C=C2)=O)C(OCC)=O)C=C1

Table S2. Central coordinates and dimensional parameters of docking grids for all target protein structures.

Target gene	PDB ID	Center coordinate (x, y, z)	Box size (Å)	Exhaustiveness
ACHE	4EY7	12.5, 30.2, 45.6	30, 30, 30	8
MDM2	1YCR	25.1, 18.3, 9.7	28, 28, 28	8
PDK1	1OKZ	52.8, 31.5, 16.2	32, 32, 32	8
RXRA	1FBY	11.3, 22.6, 38.1	30, 30, 30	8
RUNX3	1HJC	36.7, 12.4, 29.5	28, 28, 28	8
HAMP	1M4F	8.2, 15.7, 21.3	35, 35, 35	8
HAMP	2KEF	7.9, 16.1, 20.8	35, 35, 35	8

Note: All grid dimensions are presented in angstrom (Å). The exhaustiveness parameter was set to 8 for all docking calculations to balance computational efficiency and prediction accuracy.

Table S3. Full predicted binding free energies and optimal binding pose coordinates of all candidate compounds bound to six target protein structures.

Complex	Target gene	PDB ID	Compound	Binding free energy (kcal/mol)	Optimal binding pose coordinates (x, y, z)
TNF-2I47-C7	TNF	2I47	C7	-9.7	-5.189, 41.107, 45.507
TNF-3KMC-C9	TNF	3KMC	C9	-9.7	6.365, 5.677, 24.122
TNF-2I47-C9	TNF	2I47	C9	-9.6	-4.537, 40.154, 48.641
TNF-3KMC-C7	TNF	3KMC	C7	-9.5	6.053, 10.000, 23.845
TNF-3KMC-C12	TNF	3KMC	C12	-9.2	6.443, 6.034, 24.713
TNF-2I47-C6	TNF	2I47	C6	-9.1	-5.254, 40.909, 45.107
TNF-3KMC-C55	TNF	3KMC	C55	-9.1	6.321, 4.826, 24.153
TNF-3KMC-C56	TNF	3KMC	C56	-9.1	6.325, 4.932, 24.123
TNF-2I47-C1	TNF	2I47	C1	-9	-3.880, 40.483, 45.797
TNF-3KMC-C1	TNF	3KMC	C1	-9	6.578, 6.842, 25.153
TNF-2I47-C3	TNF	2I47	C3	-8.9	-3.856, 40.470, 45.807
TNF-3KMC-C5	TNF	3KMC	C5	-8.9	6.554, 6.808, 25.346
TNF-3KMC-C10	TNF	3KMC	C10	-8.9	-4.135, 23.004, 15.392
TNF-2I47-C12	TNF	2I47	C12	-8.9	-3.521, 39.837, 48.846
TNF-2I47-C56	TNF	2I47	C56	-8.9	-3.535, 39.936, 50.038
TNF-3KMC-C6	TNF	3KMC	C6	-8.8	6.320, 10.421, 23.627
TNF-2I47-C8	TNF	2I47	C8	-8.8	-5.192, 40.991, 45.279
TNF-3KMC-C3	TNF	3KMC	C3	-8.7	6.331, 9.364, 24.651
TNF-3KMC-C8	TNF	3KMC	C8	-8.7	6.086, 10.394, 23.906
TNF-2I47-C10	TNF	2I47	C10	-8.6	-3.538, 39.790, 48.796
TNF-2I47-C55	TNF	2I47	C55	-8.6	-1.781, 36.308, 45.777
TNF-2I47-C2	TNF	2I47	C2	-8.4	-5.124, 41.038, 45.571
TNF-3KMC-C2	TNF	3KMC	C2	-8.4	6.142, 10.024, 23.999
TNF-2I47-C5	TNF	2I47	C5	-8.3	-3.257, 39.867, 47.734
STAT3-6NJS-C9	STAT3	6NJS	C9	-8.2	10.004, 35.433, 20.039
HAMP-1M4F-C10	HAMP	1M4F	C10	-8.2	41.397, 42.668, 27.755
TNF-2I47-C11	TNF	2I47	C11	-8.2	-4.983, 40.894, 44.969
HAMP-1M4F-C5	HAMP	1M4F	C5	-8.1	41.381, 42.753, 27.669
HAMP-1M4F-C9	HAMP	1M4F	C9	-8.1	41.583, 42.792, 27.635
TNF-3KMC-C11	TNF	3KMC	C11	-8.1	6.194, 10.495, 24.417
TNF-2I47-C13	TNF	2I47	C13	-8.1	-4.740, 41.161, 47.026
TNF-2I47-C54	TNF	2I47	C54	-8.1	-6.989, 41.047, 45.035
HAMP-1M4F-C1	HAMP	1M4F	C1	-8	41.352, 42.756, 27.695
TNF-3KMC-C13	TNF	3KMC	C13	-8	6.145, 8.309, 23.608
TNF-3KMC-C54	TNF	3KMC	C54	-7.9	6.840, 10.550, 21.963

Complex	Target gene	PDB ID	Compound	Binding free energy (kcal/mol)	Optimal binding pose coordinates (x, y, z)
HAMP-1M4F-C3	HAMP	1M4F	C3	-7.8	41.487, 42.737, 27.692
HAMP-2KEF-C9	HAMP	2KEF	C9	-7.8	19.692, -17.288, 14.054
RELA-3CBQ-C9	RELA	3CBQ	C9	-7.7	-5.293, 14.585, 4.881
RELA-3CBQ-C10	RELA	3CBQ	C10	-7.7	-5.276, 14.706, 4.675
STAT3-6NJS-C10	STAT3	6NJS	C10	-7.6	10.042, 35.332, 20.089
HAMP-2KEF-C10	HAMP	2KEF	C10	-7.6	25.721, -13.702, 7.156
STAT3-6NJS-C12	STAT3	6NJS	C12	-7.6	0.296, 25.577, 28.542
TNF-3KMC-C49	TNF	3KMC	C49	-7.6	7.004, 4.836, 23.532
STAT3-6NJS-C5	STAT3	6NJS	C5	-7.5	0.697, 25.492, 26.610
RELA-3CBQ-C12	RELA	3CBQ	C12	-7.5	-5.396, 14.525, 4.941
TNF-2I47-C27	TNF	2I47	C27	-7.5	-4.721, 39.682, 47.440
HAMP-2KEF-C1	HAMP	2KEF	C1	-7.4	19.354, -16.961, 13.822
STAT3-6NJS-C1	STAT3	6NJS	C1	-7.4	9.377, 35.136, 20.558
HAMP-2KEF-C5	HAMP	2KEF	C5	-7.4	19.432, -16.921, 14.106
STAT3-6NJS-C7	STAT3	6NJS	C7	-7.4	-0.286, 27.616, 25.539
STAT3-6NJS-C13	STAT3	6NJS	C13	-7.4	-8.121, 29.544, 1.716
RELA-3CBQ-C1	RELA	3CBQ	C1	-7.3	-5.124, 14.690, 4.703
RELA-3CBQ-C5	RELA	3CBQ	C5	-7.3	-5.117, 14.700, 4.734
STAT3-6NJS-C56	STAT3	6NJS	C56	-7.3	11.593, 5.304, 24.178
HAMP-2KEF-C3	HAMP	2KEF	C3	-7.2	19.351, -16.938, 13.817
STAT3-6NJS-C55	STAT3	6NJS	C55	-7.2	12.217, 5.530, 24.439
TNF-2I47-C58	TNF	2I47	C58	-7.2	0.791, 37.112, 45.188
STAT3-6NJS-C3	STAT3	6NJS	C3	-7.1	0.528, 28.592, 24.824
STAT3-6NJS-C8	STAT3	6NJS	C8	-7.1	10.131, 35.876, 18.692
HAMP-1M4F-C13	HAMP	1M4F	C13	-7.1	45.875, 45.183, 23.973
STAT3-6NJS-C29	STAT3	6NJS	C29	-7.1	-8.210, 29.306, -0.412
STAT3-6NJS-C2	STAT3	6NJS	C2	-7	11.624, 34.993, 19.577
RELA-3CBQ-C3	RELA	3CBQ	C3	-7	16.181, 24.729, 15.047
STAT3-6NJS-C6	STAT3	6NJS	C6	-7	1.799, 29.078, 23.423
RELA-3CBQ-C7	RELA	3CBQ	C7	-7	-6.534, 18.605, 1.395
HAMP-2KEF-C12	HAMP	2KEF	C12	-7	20.841, -1.313, 31.792
TNF-3KMC-C38	TNF	3KMC	C38	-7	6.905, 8.966, 25.867
STAT3-6NJS-C54	STAT3	6NJS	C54	-7	12.157, 7.050, 24.769
TNF-2I47-C53	TNF	2I47	C53	-6.9	-1.036, 40.117, 45.652
RELA-3CBQ-C55	RELA	3CBQ	C55	-6.9	-5.438, 16.217, 4.263

Complex	Target gene	PDB ID	Compound	Binding free energy (kcal/mol)	Optimal binding pose coordinates (x, y, z)
RELA-3CBQ-C56	RELA	3CBQ	C56	-6.9	-6.100, 13.309, 5.445
RELA-3CBQ-C2	RELA	3CBQ	C2	-6.8	15.360, 22.780, 16.686
HAMP-2KEF-C6	HAMP	2KEF	C6	-6.8	21.993, -15.270, 11.944
HAMP-2KEF-C7	HAMP	2KEF	C7	-6.8	21.247, -15.786, 12.055
HAMP-2KEF-C8	HAMP	2KEF	C8	-6.8	20.281, -15.680, 10.883
HAMP-1M4F-C8	HAMP	1M4F	C8	-6.8	41.672, 42.503, 26.040
HAMP-2KEF-C13	HAMP	2KEF	C13	-6.8	17.340, -17.478, 12.479
TNF-2I47-C31	TNF	2I47	C31	-6.8	-2.521, 39.042, 45.885
TNF-3KMC-C37	TNF	3KMC	C37	-6.8	9.030, 8.358, 28.336
TNF-3KMC-C53	TNF	3KMC	C53	-6.8	8.047, 8.470, 26.718
STAT3-6NJS-C11	STAT3	6NJS	C11	-6.7	13.777, 8.047, 24.545
RELA-3CBQ-C13	RELA	3CBQ	C13	-6.7	15.006, 20.559, 16.432
HAMP-2KEF-C54	HAMP	2KEF	C54	-6.7	19.001, -19.407, 12.865
HAMP-1M4F-C11	HAMP	1M4F	C11	-6.6	45.838, 42.896, 23.682
HAMP-1M4F-C12	HAMP	1M4F	C12	-6.6	41.460, 43.140, 28.580
TNF-3KMC-C19	TNF	3KMC	C19	-6.6	2.531, 24.780, 17.492
TNF-3KMC-C27	TNF	3KMC	C27	-6.6	8.518, 8.400, 26.281
STAT3-6NJS-C27	STAT3	6NJS	C27	-6.6	16.813, 7.560, 21.746
TNF-2I47-C41	TNF	2I47	C41	-6.6	-2.971, 39.866, 47.010
HAMP-1M4F-C49	HAMP	1M4F	C49	-6.6	39.712, 41.946, 27.588
HAMP-1M4F-C56	HAMP	1M4F	C56	-6.6	41.920, 42.859, 29.595
TNF-3KMC-C58	TNF	3KMC	C58	-6.6	6.157, 3.863, 23.292
HAMP-2KEF-C2	HAMP	2KEF	C2	-6.5	21.106, -15.839, 12.034
HAMP-1M4F-C2	HAMP	1M4F	C2	-6.5	42.216, 42.819, 26.753
HAMP-1M4F-C7	HAMP	1M4F	C7	-6.5	30.593, 50.308, 13.263
TNF-3KMC-C29	TNF	3KMC	C29	-6.5	7.199, 9.002, 25.461
TNF-3KMC-C31	TNF	3KMC	C31	-6.5	7.272, 8.515, 26.526
TNF-3KMC-C41	TNF	3KMC	C41	-6.5	7.165, 7.269, 30.241
TNF-3KMC-C42	TNF	3KMC	C42	-6.5	5.374, 8.948, 27.651
STAT3-6NJS-C49	STAT3	6NJS	C49	-6.5	-1.891, 23.514, 27.767
HAMP-1M4F-C54	HAMP	1M4F	C54	-6.5	41.919, 42.866, 27.289
HAMP-2KEF-C55	HAMP	2KEF	C55	-6.5	22.851, -14.826, 12.272
RELA-3CBQ-C8	RELA	3CBQ	C8	-6.4	15.465, 22.686, 17.142
TNF-2I47-C32	TNF	2I47	C32	-6.4	-2.441, 39.115, 45.861
TNF-2I47-C42	TNF	2I47	C42	-6.4	-2.667, 39.375, 46.117

Complex	Target gene	PDB ID	Compound	Binding free energy (kcal/mol)	Optimal binding pose coordinates (x, y, z)
RELA-3CBQ-C54	RELA	3CBQ	C54	-6.4	-7.393, 19.372, 1.203
HAMP-2KEF-C56	HAMP	2KEF	C56	-6.4	18.166, -17.402, 16.201
RELA-3CBQ-C6	RELA	3CBQ	C6	-6.3	-6.272, 13.623, 5.680
STAT3-6NJS-C25	STAT3	6NJS	C25	-6.3	-2.705, 22.850, 28.961
TNF-3KMC-C32	TNF	3KMC	C32	-6.3	5.726, 9.028, 28.217
STAT3-6NJS-C37	STAT3	6NJS	C37	-6.3	11.909, 33.952, 19.710
HAMP-1M4F-C37	HAMP	1M4F	C37	-6.3	40.144, 41.914, 26.500
HAMP-2KEF-C11	HAMP	2KEF	C11	-6.2	20.406, -15.666, 10.895
TNF-2I47-C19	TNF	2I47	C19	-6.2	-3.268, 39.253, 53.591
HAMP-2KEF-C29	HAMP	2KEF	C29	-6.2	27.588, -14.229, 18.916
TNF-2I47-C29	TNF	2I47	C29	-6.2	-15.567, 59.276, 33.141
TNF-3KMC-C36	TNF	3KMC	C36	-6.2	7.713, 7.465, 30.607
TNF-2I47-C37	TNF	2I47	C37	-6.2	-10.660, 57.215, 33.168
STAT3-6NJS-C38	STAT3	6NJS	C38	-6.2	-7.561, 30.053, -0.571
TNF-2I47-C49	TNF	2I47	C49	-6.2	-8.868, 58.355, 38.436
STAT3-6NJS-C58	STAT3	6NJS	C58	-6.2	-1.593, 22.845, 27.913
RELA-3CBQ-C11	RELA	3CBQ	C11	-6.1	-4.607, 14.479, 4.496
TNF-3KMC-C16	TNF	3KMC	C16	-6.1	-7.218, 22.059, 16.813
HAMP-1M4F-C6	HAMP	1M4F	C6	-6	41.786, 43.334, 28.988
TNF-2I47-C17	TNF	2I47	C17	-6	-4.089, 41.388, 46.844
STAT3-6NJS-C19	STAT3	6NJS	C19	-6	15.446, 8.683, 20.478
TNF-2I47-C25	TNF	2I47	C25	-6	-12.208, 56.448, 32.224
TNF-2I47-C30	TNF	2I47	C30	-6	-1.736, 40.083, 45.572
RELA-3CBQ-C38	RELA	3CBQ	C38	-6	15.904, 20.031, 18.776
TNF-3KMC-C39	TNF	3KMC	C39	-6	6.786, 7.403, 29.714
TNF-2I47-C39	TNF	2I47	C39	-6	-0.392, 39.909, 45.206
TNF-2I47-C44	TNF	2I47	C44	-6	-1.685, 40.206, 45.580
HAMP-2KEF-C49	HAMP	2KEF	C49	-6	22.071, -14.248, 10.126
RELA-3CBQ-C49	RELA	3CBQ	C49	-6	-3.486, 14.366, 4.534
STAT3-6NJS-C53	STAT3	6NJS	C53	-6	14.555, 8.256, 21.978
HAMP-2KEF-C53	HAMP	2KEF	C53	-6	21.633, -15.262, 11.871
TNF-2I47-C16	TNF	2I47	C16	-5.9	14.293, 39.727, 29.158
TNF-3KMC-C21	TNF	3KMC	C21	-5.9	2.862, 23.251, 17.303
TNF-3KMC-C30	TNF	3KMC	C30	-5.9	9.811, 7.880, 28.851
TNF-3KMC-C35	TNF	3KMC	C35	-5.9	7.241, 8.686, 27.020

Complex	Target gene	PDB ID	Compound	Binding free energy (kcal/mol)	Optimal binding pose coordinates (x, y, z)
TNF-2I47-C35	TNF	2I47	C35	-5.9	-1.595, 39.787, 45.808
HAMP-2KEF-C37	HAMP	2KEF	C37	-5.9	23.259, -15.540, 11.734
RELA-3CBQ-C37	RELA	3CBQ	C37	-5.9	-2.889, 15.093, 4.255
HAMP-2KEF-C41	HAMP	2KEF	C41	-5.9	22.252, -13.833, 9.672
TNF-3KMC-C44	TNF	3KMC	C44	-5.9	5.992, 8.882, 27.113
TNF-2I47-C48	TNF	2I47	C48	-5.9	-3.857, 40.959, 48.984
TNF-2I47-C51	TNF	2I47	C51	-5.9	-6.171, 40.181, 46.243
TNF-3KMC-C51	TNF	3KMC	C51	-5.9	7.045, 9.864, 22.426
TNF-3KMC-C17	TNF	3KMC	C17	-5.8	8.686, 7.956, 29.083
HAMP-2KEF-C19	HAMP	2KEF	C19	-5.8	20.336, -15.921, 12.579
TNF-3KMC-C24	TNF	3KMC	C24	-5.8	-4.213, 22.701, 15.096
RELA-3CBQ-C27	RELA	3CBQ	C27	-5.8	-7.795, 17.346, 3.413
HAMP-1M4F-C55	HAMP	1M4F	C55	-5.8	41.898, 42.586, 24.992
TNF-2I47-C24	TNF	2I47	C24	-5.7	-10.411, 55.462, 32.974
TNF-2I47-C26	TNF	2I47	C26	-5.7	-4.373, 41.774, 48.820
RELA-3CBQ-C29	RELA	3CBQ	C29	-5.7	9.011, 27.545, 0.912
TNF-2I47-C33	TNF	2I47	C33	-5.7	-0.381, 39.880, 45.208
TNF-2I47-C36	TNF	2I47	C36	-5.7	-0.941, 37.425, 45.585
TNF-2I47-C38	TNF	2I47	C38	-5.7	-6.238, 49.377, 50.015
HAMP-2KEF-C16	HAMP	2KEF	C16	-5.6	21.341, -15.585, 11.592
HAMP-2KEF-C17	HAMP	2KEF	C17	-5.6	21.599, -15.635, 12.039
TNF-2I47-C21	TNF	2I47	C21	-5.6	-9.751, 59.277, 38.882
TNF-3KMC-C26	TNF	3KMC	C26	-5.6	5.007, 8.920, 21.463
STAT3-6NJS-C31	STAT3	6NJS	C31	-5.6	8.777, 29.629, -1.693
TNF-3KMC-C33	TNF	3KMC	C33	-5.6	9.407, 7.930, 27.728
STAT3-6NJS-C16	STAT3	6NJS	C16	-5.5	13.933, 9.536, 22.801
HAMP-1M4F-C17	HAMP	1M4F	C17	-5.5	41.721, 42.678, 27.730
STAT3-6NJS-C24	STAT3	6NJS	C24	-5.5	15.007, 7.039, 22.082
TNF-3KMC-C25	TNF	3KMC	C25	-5.5	3.670, 20.214, 45.566
TNF-3KMC-C28	TNF	3KMC	C28	-5.5	-3.577, 22.906, 14.954
TNF-2I47-C28	TNF	2I47	C28	-5.5	-12.793, 55.810, 34.014
HAMP-1M4F-C38	HAMP	1M4F	C38	-5.5	47.892, 43.639, 22.773
RELA-3CBQ-C41	RELA	3CBQ	C41	-5.5	15.215, 19.467, 16.707
STAT3-6NJS-C41	STAT3	6NJS	C41	-5.5	12.720, 8.625, 26.162
HAMP-2KEF-C58	HAMP	2KEF	C58	-5.5	26.757, -10.473, 3.906

Complex	Target gene	PDB ID	Compound	Binding free energy (kcal/mol)	Optimal binding pose coordinates (x, y, z)
RELA-3CBQ-C17	RELA	3CBQ	C17	-5.4	14.211, 24.590, 15.896
HAMP-2KEF-C27	HAMP	2KEF	C27	-5.4	27.368, -14.302, 6.857
HAMP-1M4F-C29	HAMP	1M4F	C29	-5.4	39.160, 33.014, 3.623
HAMP-1M4F-C42	HAMP	1M4F	C42	-5.4	35.021, 32.136, 11.603
STAT3-6NJS-C51	STAT3	6NJS	C51	-5.4	-7.479, 29.910, -0.610
RELA-3CBQ-C53	RELA	3CBQ	C53	-5.4	15.475, 22.123, 17.622
STAT3-6NJS-C17	STAT3	6NJS	C17	-5.3	10.142, 35.681, 18.209
HAMP-1M4F-C25	HAMP	1M4F	C25	-5.3	24.473, 30.408, 18.101
STAT3-6NJS-C26	STAT3	6NJS	C26	-5.3	11.415, 34.663, 18.832
HAMP-1M4F-C27	HAMP	1M4F	C27	-5.3	25.014, 40.996, 26.398
RELA-3CBQ-C31	RELA	3CBQ	C31	-5.3	3.483, 27.075, -5.802
STAT3-6NJS-C48	STAT3	6NJS	C48	-5.3	10.808, 35.558, 17.847
HAMP-1M4F-C53	HAMP	1M4F	C53	-5.3	26.939, 50.162, 15.742
RELA-3CBQ-C58	RELA	3CBQ	C58	-5.3	12.424, 26.120, 6.757
RELA-3CBQ-C19	RELA	3CBQ	C19	-5.2	14.627, 24.231, 15.647
RELA-3CBQ-C25	RELA	3CBQ	C25	-5.2	13.396, 25.204, 4.170
HAMP-2KEF-C25	HAMP	2KEF	C25	-5.2	23.048, -4.812, 33.346
STAT3-6NJS-C30	STAT3	6NJS	C30	-5.2	13.697, 9.491, 24.714
HAMP-2KEF-C38	HAMP	2KEF	C38	-5.2	28.553, -13.922, 19.497
TNF-3KMC-C48	TNF	3KMC	C48	-5.2	6.260, 12.827, 23.185
HAMP-1M4F-C58	HAMP	1M4F	C58	-5.2	29.847, 52.151, 15.418
RELA-3CBQ-C16	RELA	3CBQ	C16	-5.1	-5.968, 17.716, 2.087
STAT3-6NJS-C21	STAT3	6NJS	C21	-5.1	-1.312, 24.153, 29.972
STAT3-6NJS-C28	STAT3	6NJS	C28	-5.1	11.685, 4.113, 40.653
HAMP-1M4F-C30	HAMP	1M4F	C30	-5.1	32.473, 30.900, 9.713
RELA-3CBQ-C32	RELA	3CBQ	C32	-5.1	3.872, 26.155, -4.296
HAMP-2KEF-C36	HAMP	2KEF	C36	-5.1	21.940, -13.809, 9.605
STAT3-6NJS-C39	STAT3	6NJS	C39	-5.1	-6.878, 31.363, -0.248
TNF-2I47-C52	TNF	2I47	C52	-5.1	-2.188, 39.432, 47.822
HAMP-1M4F-C16	HAMP	1M4F	C16	-5	40.721, 42.242, 26.833
HAMP-1M4F-C21	HAMP	1M4F	C21	-5	41.367, 42.675, 27.982
HAMP-1M4F-C26	HAMP	1M4F	C26	-5	27.342, 48.609, 13.829
HAMP-2KEF-C32	HAMP	2KEF	C32	-5	23.158, -14.340, 11.763
RELA-3CBQ-C39	RELA	3CBQ	C39	-5	4.662, 26.779, -3.499
RELA-3CBQ-C42	RELA	3CBQ	C42	-5	15.317, 19.651, 16.637

Complex	Target gene	PDB ID	Compound	Binding free energy (kcal/mol)	Optimal binding pose coordinates (x, y, z)
HAMP-2KEF-C42	HAMP	2KEF	C42	-5	22.317, -13.888, 9.666
TNF-3KMC-C52	TNF	3KMC	C52	-5	5.599, 6.427, 24.564
HAMP-1M4F-C19	HAMP	1M4F	C19	-4.9	38.330, 43.527, 30.611
HAMP-1M4F-C24	HAMP	1M4F	C24	-4.9	29.172, 28.829, 15.230
RELA-3CBQ-C24	RELA	3CBQ	C24	-4.9	18.464, 16.399, -14.509
RELA-3CBQ-C28	RELA	3CBQ	C28	-4.9	0.114, 7.250, -6.234
RELA-3CBQ-C30	RELA	3CBQ	C30	-4.9	3.449, 27.351, -6.000
HAMP-1M4F-C31	HAMP	1M4F	C31	-4.9	32.214, 31.758, 10.383
HAMP-2KEF-C31	HAMP	2KEF	C31	-4.9	23.030, -14.072, 11.738
STAT3-6NJS-C32	STAT3	6NJS	C32	-4.9	9.791, 28.536, -0.721
RELA-3CBQ-C33	RELA	3CBQ	C33	-4.9	3.850, 26.025, -4.184
STAT3-6NJS-C36	STAT3	6NJS	C36	-4.9	7.088, 36.131, 19.204
RELA-3CBQ-C48	RELA	3CBQ	C48	-4.9	-2.963, 13.733, 4.432
HAMP-1M4F-C51	HAMP	1M4F	C51	-4.9	38.592, 34.503, 4.130
HAMP-2KEF-C26	HAMP	2KEF	C26	-4.8	28.294, -15.003, 18.576
HAMP-1M4F-C28	HAMP	1M4F	C28	-4.8	45.894, 44.218, 25.342
HAMP-1M4F-C33	HAMP	1M4F	C33	-4.8	35.063, 32.151, 11.567
RELA-3CBQ-C36	RELA	3CBQ	C36	-4.8	-4.409, 16.618, 3.912
HAMP-1M4F-C41	HAMP	1M4F	C41	-4.8	43.169, 42.288, 25.357
STAT3-6NJS-C42	STAT3	6NJS	C42	-4.8	15.500, 9.242, 21.120
RELA-3CBQ-C44	RELA	3CBQ	C44	-4.8	3.334, 26.022, -5.007
STAT3-6NJS-C44	STAT3	6NJS	C44	-4.8	-10.030, 0.677, 28.112
HAMP-2KEF-C48	HAMP	2KEF	C48	-4.7	18.287, -16.607, 11.353
STAT3-6NJS-C52	STAT3	6NJS	C52	-4.7	10.538, 34.042, 21.542
RELA-3CBQ-C21	RELA	3CBQ	C21	-4.6	15.846, 20.010, 18.739
HAMP-2KEF-C24	HAMP	2KEF	C24	-4.6	26.296, -13.354, 4.330
HAMP-2KEF-C28	HAMP	2KEF	C28	-4.6	20.130, -19.358, 14.082
HAMP-2KEF-C30	HAMP	2KEF	C30	-4.6	23.229, -13.691, 10.499
HAMP-1M4F-C32	HAMP	1M4F	C32	-4.6	43.936, 42.394, 24.640
HAMP-2KEF-C33	HAMP	2KEF	C33	-4.6	22.210, -13.858, 9.685
STAT3-6NJS-C33	STAT3	6NJS	C33	-4.6	-7.539, 30.244, -0.826
HAMP-2KEF-C44	HAMP	2KEF	C44	-4.6	20.350, -15.558, 10.560
HAMP-1M4F-C48	HAMP	1M4F	C48	-4.6	27.141, 33.838, 16.618
HAMP-2KEF-C52	HAMP	2KEF	C52	-4.6	19.570, -15.617, 10.258
RELA-3CBQ-C26	RELA	3CBQ	C26	-4.5	11.953, 3.145, 9.508

Complex	Target gene	PDB ID	Compound	Binding free energy (kcal/mol)	Optimal binding pose coordinates (x, y, z)
STAT3-6NJS-C35	STAT3	6NJS	C35	-4.5	7.908, 35.249, 21.136
RELA-3CBQ-C35	RELA	3CBQ	C35	-4.5	-4.381, 12.913, 5.007
RELA-3CBQ-C51	RELA	3CBQ	C51	-4.5	15.272, -2.207, 8.139
RELA-3CBQ-C52	RELA	3CBQ	C52	-4.5	-3.339, 13.529, 4.710
HAMP-2KEF-C35	HAMP	2KEF	C35	-4.4	30.280, -16.082, 19.538
HAMP-1M4F-C36	HAMP	1M4F	C36	-4.4	46.667, 42.265, 22.830
HAMP-1M4F-C39	HAMP	1M4F	C39	-4.4	41.156, 27.808, 24.502
HAMP-2KEF-C39	HAMP	2KEF	C39	-4.4	27.070, -13.005, 19.372
HAMP-2KEF-C51	HAMP	2KEF	C51	-4.3	18.981, -19.461, 15.838
HAMP-2KEF-C21	HAMP	2KEF	C21	-4.2	38.835, -0.960, 30.698
HAMP-1M4F-C35	HAMP	1M4F	C35	-4.2	54.281, 30.912, 16.667
HAMP-1M4F-C44	HAMP	1M4F	C44	-4.2	25.594, 40.970, 23.332
HAMP-1M4F-C52	HAMP	1M4F	C52	-4.2	53.541, 29.692, 16.197

Note: All binding free energies were calculated via AutoDock Vina software, with units of kcal/mol. The table lists the complete docking results of 43 candidate compounds across 6 target protein structures, and only the optimal lowest-energy conformation for each compound–receptor pair is presented.

Table S4. Protein–ligand interaction analysis of compound 16 and compound 21 with HAMP (PDB: 1M4F, 2KEF) by PLIP.

Complex	Interaction type	Residues / details
1M4F–16	Hydrophobic contacts	PRO5, ILE6, ILE8, MET21
1M4F–16	Hydrogen bonds	ILE6, CYS7, ILE8, CYS23
2KEF–16	Hydrophobic contacts	ASP1, HIS3, PHE4
2KEF–16	H-bonds / π-Cation	PHE4 (H-bond), HIS3 (π-Cation)

Note: Non-covalent interactions were analyzed by Protein–Ligand Interaction Profiler (PLIP, https://plip-tool.biotec.tu-dresden.de/).

Table S5. Purity, NMR, and HR-MS data for compounds 16 and 21.

Property	Compound 16	Compound 21
Name	[6]-shogaol	1-(4-hydroxy-3-methoxyphenyl)-5-

		methoxydecan-3-one
Formula	C17H24O3	C18H28O4
HR-ESI-MS	m/z 277 [M+H]+	m/z 307 [M-H]−

Note: NMR and HR-MS data were originally reported in Liu QR. Master's thesis: Phenylpropanoid Amides from Buteae Monospermae Semen and their anti-HCoV-OC43 activity. Yunnan University of Chinese Medicine, 2024. Full ^{1}H/^{13}C NMR data are provided in the original isolation reports. Compound 16 was identified as [6]-shogaol by spectral comparison [43].

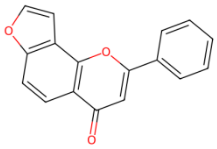
C1

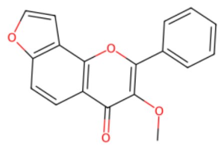
C2

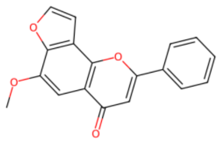
C3

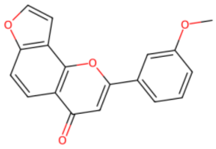
C5

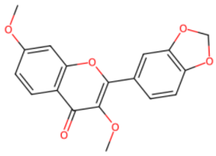
C6

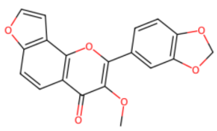
C7

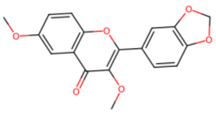
C8

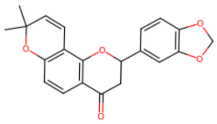
C9

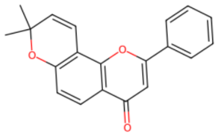
C10

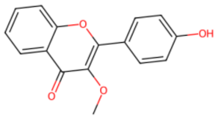
C11

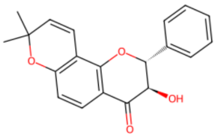
C12

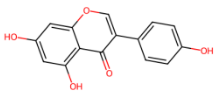
C13

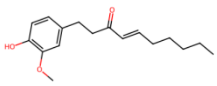
C16

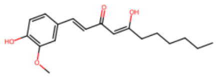
C17

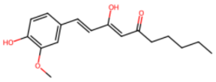
C19

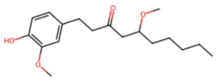
C21

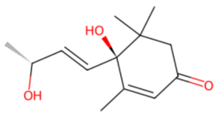
C24

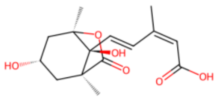
C25

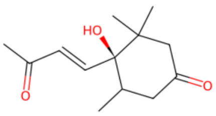
C26

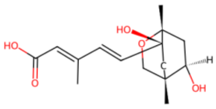
C27

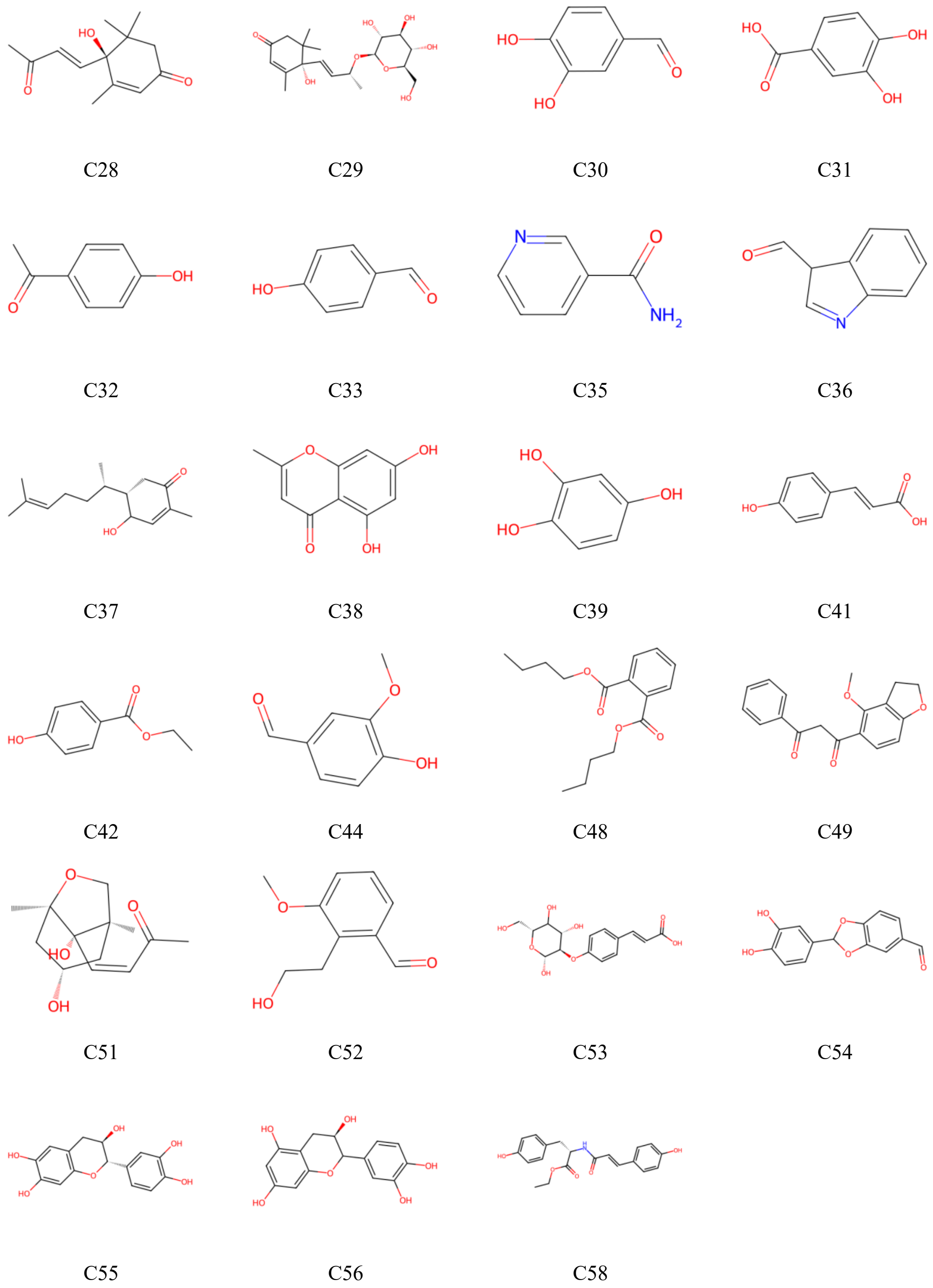

Figure S1. Two-dimensional chemical structures of all 43 BMS-derived candidate compounds.

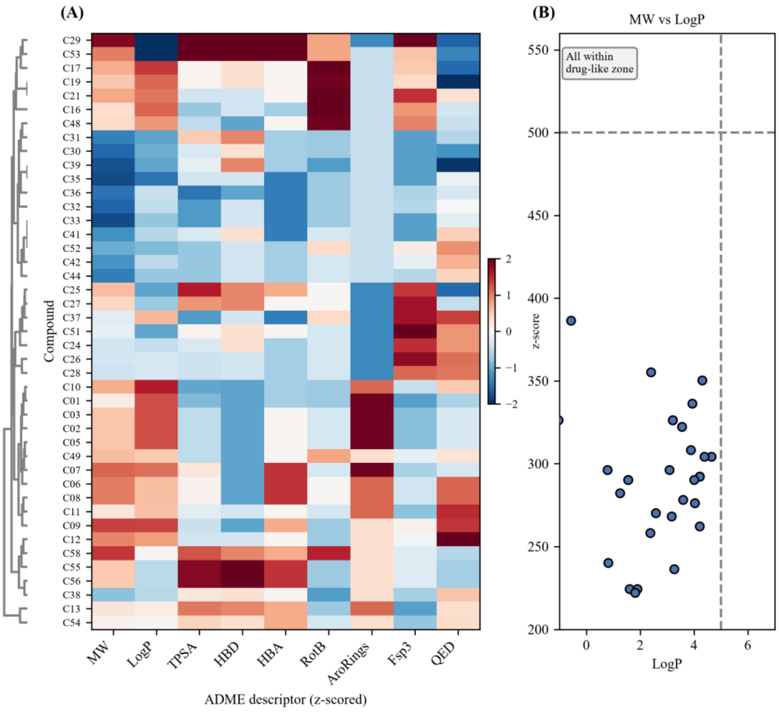

Figure S2. Summary of ADMET screening results for the 43 ADMET-screened BMS compounds. (A) Heatmap of RDKit-computed ADME descriptors (molecular weight, LogP, topological polar surface area, hydrogen bond donors, hydrogen bond acceptors, rotatable bonds, aromatic rings, fraction of sp3 carbons, and quantitative estimate of drug-likeness) z-scored across compounds and hierarchically clustered by Euclidean distance. (B) Scatter plot of molecular weight versus LogP; dashed lines denote Lipinski thresholds (molecular weight = 500 Da, LogP = 5). All 43 compounds fall within the drug-like chemical space. ADMET, absorption, distribution, metabolism, excretion, and toxicity; BMS, Buteae Monospermae Semen; LogP, octanol–water partition coefficient; QED, quantitative estimate of drug-likeness; TPSA, topological polar surface area.

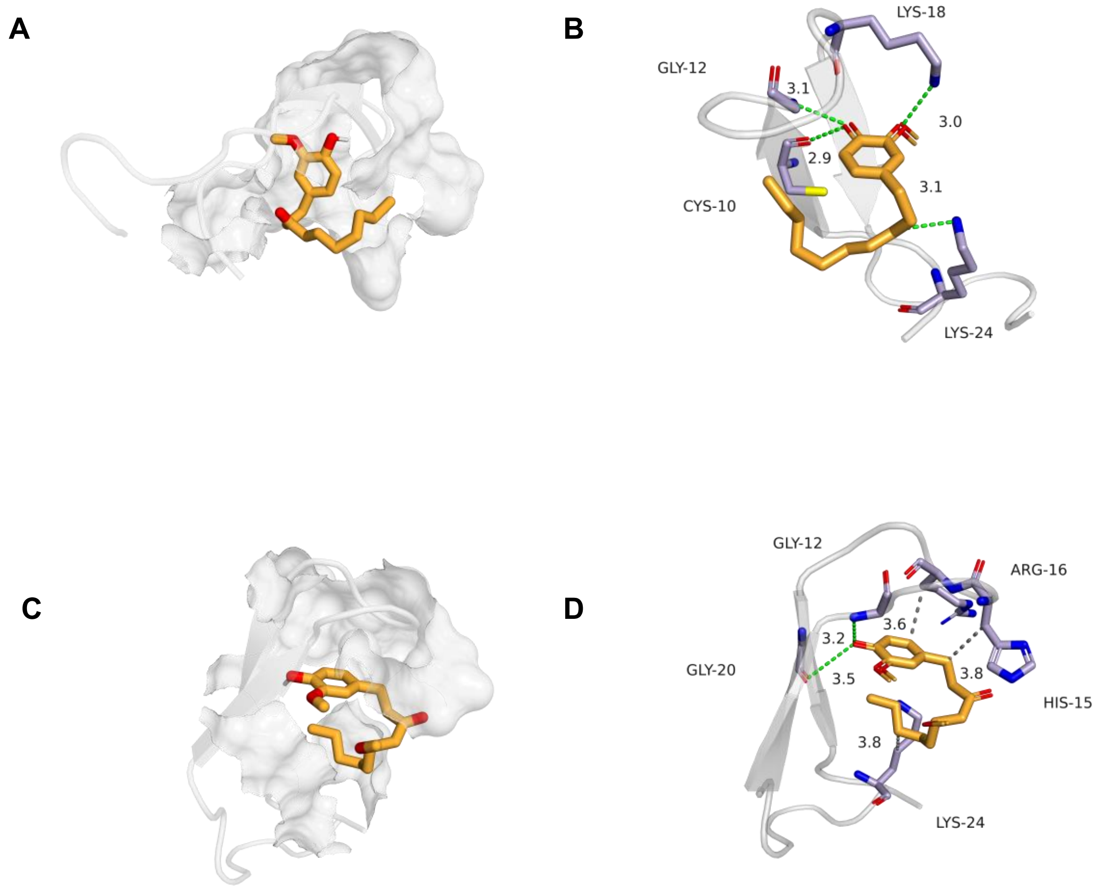

Figure S3. Molecular docking interaction profiles of compound **16** and **c**ompound **21** complexed with HAMP (PDB ID: 1M4F). (A) Overall binding pocket conformation of compound **16** embedded in the surface cavity of HAMP (1M4F). (B) Detailed two-dimensional intermolecular interaction diagram showing hydrogen bonds and hydrophobic contacts between compound **16** and surrounding amino acid residues, with labeled bond distances (Å). (C) Global binding conformation of compound **21** within the HAMP pocket of the 1M4F structure. (D) Schematic of key non-covalent interactions formed between compound **21** and the adjacent residues of HAMP (1M4F), with hydrogen bond lengths marked. HAMP, hepcidin antimicrobial peptide.

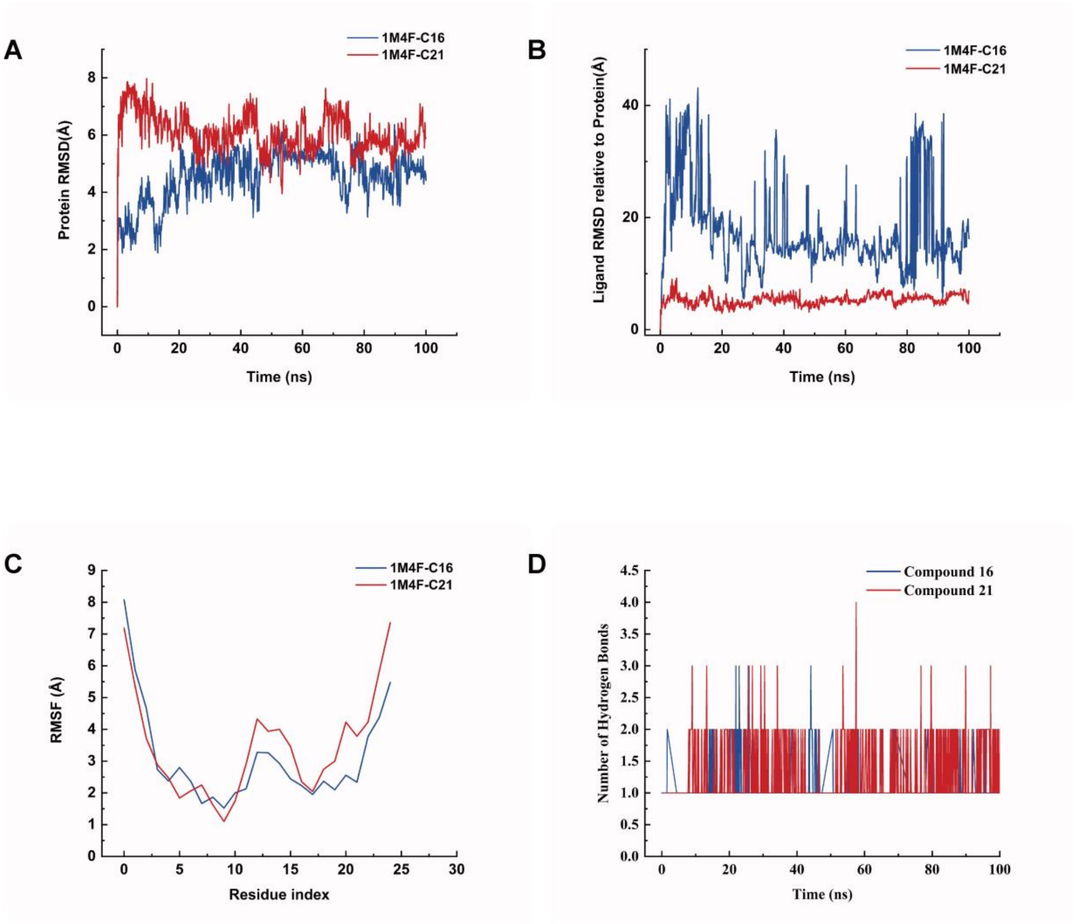

Figure S4. Molecular dynamics trajectory analysis of compound **16** and **compound 21** complexed with HAMP (PDB ID: 1M4F). (A) Backbone root-mean-square deviation (RMSD) curves of the HAMP protein (1M4F) bound to compound **16** and compound **21** throughout the 100 ns simulation. (B) Ligand RMSD relative to the initial docking conformation for the 1M4F–16 and 1M4F–21 systems. (C) Root-mean-square fluctuation (RMSF) distribution of amino acid residues of HAMP (1M4F) complexed with the two lead compounds, reflecting residue flexibility. (D) Time-dependent variation of intermolecular hydrogen bond counts between HAMP (1M4F) and compound **16**/21; raw and smoothed curves are provided for comparison. RMSD, root-mean-square deviation; RMSF, root-mean-square fluctuation.

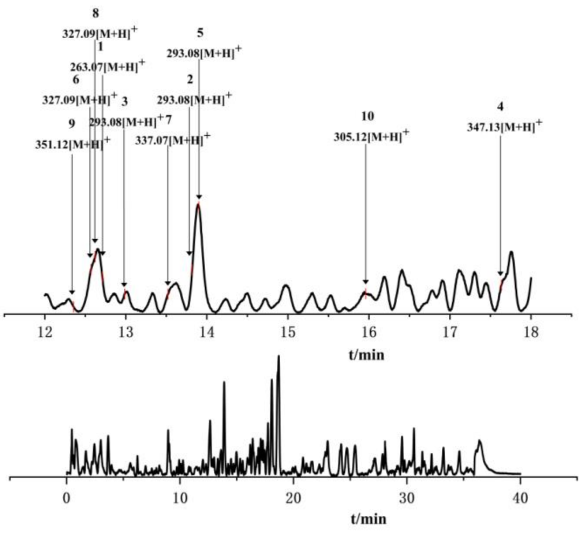

Figure S5. LC–MS chemical fingerprint of the ethyl acetate extract of *Buteae Monospermae Semen*. Total ion chromatogram (bottom) and extracted ion chromatograms of compounds **1** and **6** with the corresponding [M+H]+ ions (top) are shown.

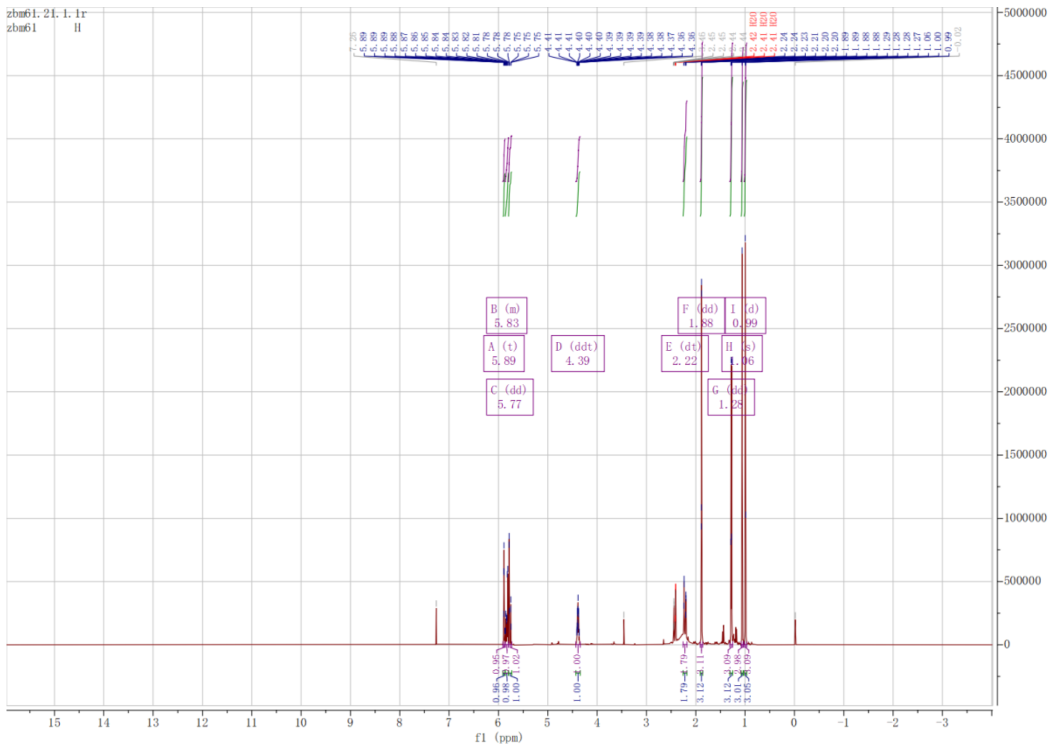

(A) Compound 16, ¹H NMR (500 MHz, $CDCl_3$).

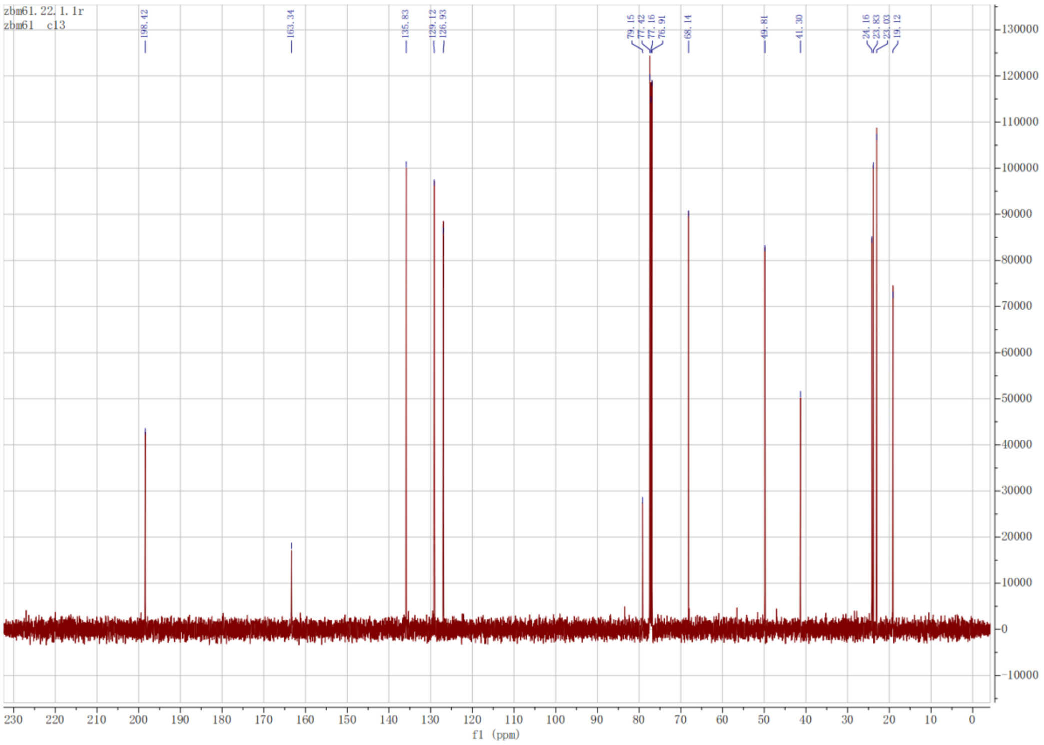

(B) Compound 16, ^{13}C NMR (126 MHz, $CDCl_3$).

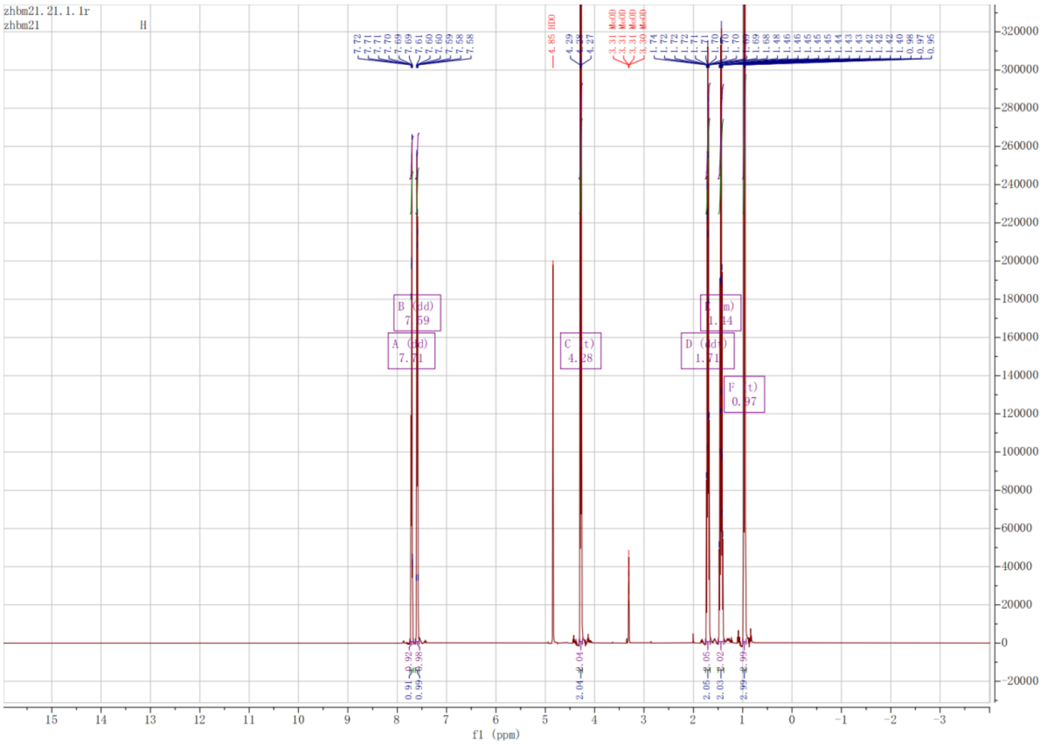

(C) Compound 21, ^{1}H NMR (500 MHz, $CDCl_3$).

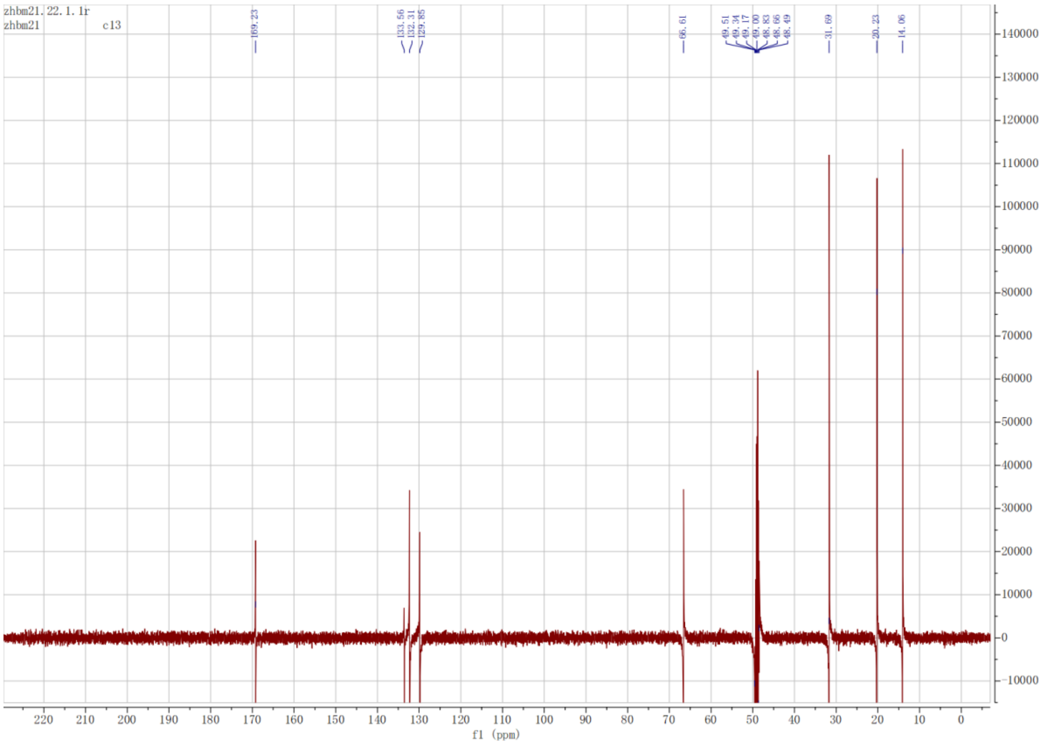

(D) Compound 21, ^{13}C NMR (126 MHz, $CDCl_3$).

Note: NMR spectra were measured in $CDCl_3$ at 25 °C. Raw spectral data are from the authors' laboratory records.

Figure S6. NMR spectra of compounds 16 and 21.
